# Modeling cognition through adaptive neural synchronization: a multimodal framework using EEG, fMRI, and reinforcement learning

**DOI:** 10.3389/fncom.2025.1616472

**Published:** 2025-10-16

**Authors:** Rashad Hall, Maury Jackson, Maryam Maleki, Horace T. Crogman

**Affiliations:** ^1^Department of Physics, California State University, Dominguez Hills, Carson, CA, United States; ^2^H.M.S Richard Divinity School, La Sierra University, Riverside, CA, United States

**Keywords:** reinforcement learning, neuronal synchronization, EEG-fMRI integration, Kuramoto oscillator, cognitive modeling, energy-efficient computation, brain-inspired AI, Q-learning

## Abstract

**Introduction:**

Understanding the cognitive process of thinking as a neural phenomenon remains a central challenge in neuroscience and computational modeling. This study addresses this challenge by presenting a biologically grounded framework that simulates adaptive decision making across cognitive states.

**Methods:**

The model integrates neuronal synchronization, metabolic energy consumption, and reinforcement learning. Neural synchronization is simulated using Kuramoto oscillators, while energy dynamics are constrained by multimodal activity profiles. Reinforcement learning agents—Q-learning and Deep Q-Network (DQN)—modulate external inputs to maintain optimal synchrony with minimal energy cost. The model is validated using real EEG and fMRI data, comparing simulated and empirical outputs across spectral power, phase synchrony, and BOLD activity.

**Results:**

The DQN agent achieved rapid convergence, stabilizing cumulative rewards within 200 episodes and reducing mean synchronization error by over 40%, outperforming Q-learning in speed and generalization. The model successfully reproduced canonical brain states—focused attention, multitasking, and rest. Simulated EEG showed dominant alpha-band power (3.2 × 10^−4^ a.u.), while real EEG exhibited beta-dominance (3.2 × 10^−4^ a.u.), indicating accurate modeling of resting states and tunability for active tasks. Phase Locking Value (PLV) ranged from 0.9806 to 0.9926, with the focused condition yielding the lowest circular variance (0.0456) and a near significant phase shift compared to rest (*t* = −2.15, *p* = 0.075). Cross-modal validation revealed moderate correlation between simulated and real BOLD signals (*r* = 0.30, resting condition), with delayed inputs improving temporal alignment. General Linear Model (GLM) analysis of simulated BOLD data showed high region-specific prediction accuracy (*R*^2^ = 0.973–0.993, *p* < 0.001), particularly in prefrontal, parietal, and anterior cingulate cortices. Voxel-wise correlation and ICA decomposition confirmed structured network dynamics.

**Discussion:**

These findings demonstrate that the framework captures both electrophysiological and spatial aspects of brain activity, respects neuroenergetic constraints, and adaptively regulates brain-like states through reinforcement learning. The model offers a scalable platform for simulating cognition and developing biologically inspired neuroadaptive systems.

**Conclusion:**

This work provides a novel and testable approach to modeling thinking as a biologically constrained control problem and lays the groundwork for future applications in cognitive modeling and brain-computer interfaces.

## 1 Introduction

Thinking, a complex and dynamic process underpinning human cognition, emerges from the intricate interplay of neural activity, sensory inputs, and energy dynamics. This interplay not only enables coherent thought but also facilitates adaptive decision-making and learning. Recent advances in neuroscience have highlighted the importance of neuronal synchronization where neurons fire in coordinated patterns—as a key mechanism underlying cognitive focus and mental coherence. However, the brain must strike a delicate balance between maintaining this synchronization and conserving metabolic resources, as neural activity demands significant energy expenditure (Pinker, 2011; Raichle, 2010).

Modern neuroimaging tools, such as electroencephalography (EEG) and functional magnetic resonance imaging (fMRI), provide unprecedented insights into the neural correlates of thinking by capturing spatiotemporal patterns of brain activity. These technologies reveal how neuronal networks synchronize during tasks requiring attention, learning, and decision-making. Despite these advances, there remains a lack of an integrated mathematical framework that combines neuronal synchronization, energy consumption, and adaptive behavior to model the thinking process comprehensively (Neurolaunch, n.d.).

This study proposes a novel framework to bridge this gap by:

1. Modeling neuronal synchronization as a quantifiable measure of cognitive focus, drawing upon the Kuramoto model for coupled oscillatory systems (Strogatz, 2000).
2. Quantifying energy dynamics in cognitive processes using neuroimaging data, particularly EEG and fMRI, to map transitions between cognitive states.
3. Implementing reinforcement learning through Q-learning to simulate adaptive decision-making, where the brain learns to optimize cognitive states for efficiency and task performance.

By integrating these elements, the proposed framework seeks to replicate key features of human thinking through simulations, offering new avenues for understanding and optimizing cognitive function. The potential applications of this model range from enhancing educational methodologies to developing neuroadaptive technologies and addressing cognitive disorders.

The integration of concepts from quantum psychology also informs this framework, emphasizing the non-linear, probabilistic nature of cognitive states and their transitions. This perspective suggests that human thought processes might share parallels with quantum systems, wherein decision-making and cognitive shifts occur within complex, multidimensional landscapes (Neurolaunch, n.d.). These insights provide an additional layer to the exploration of thinking, underscoring the need for interdisciplinary approaches to unravel the mysteries of human cognition. To model thinking in a biologically realistic way, it is essential to integrate mechanisms of neuronal synchronization, adaptive control, and metabolic energy constraints. The brain continuously balances these dimensions—organizing neural activity (synchronization), responding to stimuli (adaptation), and preserving efficiency (energy use). This study seeks to capture that balance by combining dynamic synchronization modeling, reinforcement learning, and real neurophysiological data into a unified simulation of cognitive state transitions.

## 2 Literature review

Understanding human thinking as a process of neuronal synchronization influenced by external stimuli and cognitive demands has been a focal point in neuroscience and computational modeling. Research has demonstrated that neuronal synchronization, particularly in specific frequency bands such as alpha (8–12 Hz) and gamma (30–100 Hz), plays a critical role in cognitive functions like attention, memory, and perception (Garrett et al., 2024; Gupta and Bahmer, 2021; Scheeringa et al., 2011). Functional magnetic resonance imaging (fMRI) studies have shown that as cognitive tasks become more difficult, neural activity—especially in the prefrontal cortex—increases, indicating heightened mental engagement (Crick, 1996; Pagnoni et al., 2008; Kuhl, 2010; Nani et al., 2019; Dai, 2024). This association between neural activation and mental processing supports the idea that thinking can be tracked through patterns of brain activity.

A useful analogy compares this to the random motion of electrons in a metal. When an electric field is applied, electrons align in a specific direction. Similarly, neurons in the brain fire randomly until external stimuli—combined with memory and attention—cause them to synchronize (Crogman and Jackson, 2023). This synchronization marks the emergence of focused thought. For example, when a person sees something that captures their interest, their attention shifts, and a new thought process begins. In this framework, multitasking is less about simultaneous thought and more about rapid shifts in focus driven by competing stimuli (Crogman and Jackson, 2023).

Synchronization facilitates communication between brain regions, enabling efficient information processing. Computational models, such as those based on the Kuramoto oscillator, have been employed to simulate synchronization dynamics in neural populations (Kuramoto, 1975). Recent work has further emphasized how local and global synchrony govern attention-related cortical activation (Gundlach et al., 2024). However, while these studies underscore the importance of synchronization, they often do not address the emergence of this coherence from stochastic, diffuse neural states, particularly in the context of human thinking (Fries, 2005; Singer, 1999; Breakspear et al., 2010).

The brain is frequently conceptualized as a noisy system, with random neural firings providing a foundation for its dynamical behavior. Studies have explored how stochastic neural activity can transition into coherent states driven by task demands or external influences. Deco et al. (2009) discussed how these transitions underpin dynamic brain states, while Beggs and Plenz (2003) suggested that the brain operates near criticality, where the balance between noise and order optimizes computational efficiency. More recent findings also suggest that shifts in cortical synchrony can mark transitions into different states of awareness or readiness for action (Arzate-Mena et al., 2022). Despite these insights, explicit modeling of cognitive states and their transitions from randomness to synchronization remains underdeveloped.

Physical analogies have long been used to describe neural systems. For instance, models liken synchronization in the brain to physical systems such as magnetization in the Ising model or coupled oscillators in dynamic systems (Hopfield, 1982; Haken, 1983). These analogies illustrate how external forces, such as sensory input or intent, guide independent systems toward coherence. However, the specific application of these physical analogies to model human thinking as transitions between random and synchronized neural states is relatively unexplored.

Reinforcement learning has also been employed to simulate decision-making and adaptive behavior in neural systems. These models focus on optimizing actions based on rewards but typically do not incorporate the underlying synchronization dynamics or energy efficiency during cognitive transitions (Sutton and Barto, 1998). More recently, deep reinforcement learning has been used to decode and respond to attention states in real-time, demonstrating feasibility for adaptive brain-computer interfaces (Rehman et al., 2025; Botvinick et al., 2020). While reinforcement learning excels at simulating adaptive processes, it has yet to be integrated with neural synchronization and energy modeling to fully capture the complexity of thinking.

This study builds upon these foundations to develop a novel computational framework that integrates synchronization dynamics, energy consumption, and reinforcement learning to simulate human thinking. This framework models thinking as a transition from random, diffuse neural states to synchronized patterns of activity. Using the Kuramoto model, neurons are treated as coupled oscillators, with synchronization representing unified cognitive states. Unlike prior studies, this approach explicitly simulates how random neural activity aligns under the influence of external stimuli, memory recall, or intent. Furthermore, it incorporates energy dynamics by utilizing EEG and fMRI data to quantify the metabolic cost of transitioning between cognitive states, addressing gaps in prior work that have largely considered synchronization and energy efficiency in isolation (Schoknecht et al., 2025; Universität Leipzig, 2025; Zandt et al., 2011; Raichle, 2010).

To simulate adaptive thinking, the framework employs Q-learning, a form of reinforcement learning, to optimize synchronization and energy use during cognitive transitions. The Q-learning model links synchronization dynamics and energy consumption to rewards, providing a novel method for simulating the adaptive nature of human thought. Recent research has shown that reinforcement learning models applied to EEG and spiking neural networks can capture task dynamics and delay-adaptive behaviors in biologically plausible ways (Nadafian et al., 2024; Zhang et al., 2024). Additionally, reinforcement learning has been shown to operate effectively near the edge of synchronization transitions, which is precisely the regime modeled by the Kuramoto framework (Khoshkhou and Montakhab, 2022). By drawing on publicly available EEG and fMRI datasets for validation, this study ensures robust and reproducible results, distinguishing itself from purely theoretical models. The use of interdisciplinary analogies—such as electron alignment or magnetic domains—further enriches the conceptual understanding of synchronization, emphasizing its universality across complex systems.

This framework advances the study of human cognition by integrating neuronal synchronization, energy modeling, and adaptive decision-making into a unified computational paradigm. It addresses gaps in existing research by simulating transitions from random neural states to focused cognitive states and exploring the energy efficiency of these processes. Recent reviews highlight the importance of such integrative models in computational neuroimaging and emphasize that combining oscillations, mutual information, and synchrony will be critical for the next generation of brain-inspired models (Dai, 2024; Loosen et al., 2024). This work has significant implications for understanding cognitive processes, including attention, multitasking, and disorders of thought. While prior studies have independently examined neuronal synchrony, energy modeling, or reinforcement learning in neural contexts, our work uniquely integrates all three. The novelty of this study lies in the synthesis of Kuramoto-based synchrony modeling, an energy cost function informed by EEG and fMRI, and biologically constrained reinforcement learning. This allows us to simulate how cognitive states evolve dynamically under task and energy demands—an area not fully explored in existing models.

## 3 Methods

This study employs publicly available datasets and computational modeling to explore the dynamics of thinking, focusing on the interplay between neuronal synchronization, energy consumption, and adaptive decision-making. The methodology involves three main phases: acquiring and preprocessing neuroimaging data, developing computational models, and conducting simulations to validate the framework. The description of the Kuramoto oscillator modeling, reinforcement learning integration, and reward function design was initially written by the authors and partially edited using OpenAI ChatGPT (GPT-4, April 2024) to enhance clarity. All underlying algorithms, equations, and simulation results were developed and validated independently by the authors. The AI tool did not contribute to the scientific interpretation, data analysis, or experimental design.

### 3.1 Theoretical framework

Human thinking can be viewed as a process of neural synchronization, transitioning from stochastic, diffuse activity to coherent, focused states. At its core, this process can be modeled as a system of coupled oscillators. Neurons, operating at their natural frequencies, represent random background activity when uncoordinated. However, during focused thought, external forces such as sensory input or intentional focus act to synchronize these neurons, creating a coherent frequency that represents unified cognition.

This phenomenon is analogous to physical systems in which independent components align under external influence. For example, electrons align in response to an electric field, and magnetic domains synchronize under external magnetization (Crogman and Jackson, 2023). Similarly, in the brain, sensory stimuli or task demands act as forces that drive neurons into alignment, forming the neural basis of focused thought. By employing the Kuramoto model of coupled oscillators, we mathematically represent this transition, exploring how synchronization emerges and is maintained.

Through this approach, the study aims to capture the underlying principles of human cognition, offering insights into how the brain organizes immense neural complexity into coherent states. Furthermore, modeling these transitions provides a foundation for understanding not only normal cognitive processes such as attention and multitasking but also pathological conditions where synchronization may be disrupted, such as in ADHD, schizophrenia, or other cognitive disorders.

**Figure 1:**
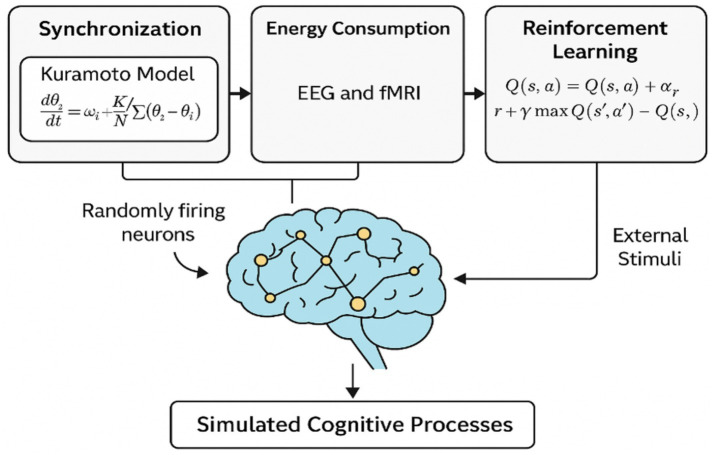
Conceptual framework for modeling cognition as synchronized neuronal dynamics. Random firing evolves into organized brain states via the Kuramoto model, with EEG/fMRI features guiding a reinforcement learning agent to optimize external input based on energy and synchrony.

Figure 1 presents an overview of the theoretical model underlying this study. At the core of the framework is the hypothesis that thinking emerges from initially random neuronal firing that becomes synchronized in response to external stimuli. We represent this synchronization using the Kuramoto model of coupled oscillators, allowing us to simulate collective neural dynamics. The resulting signals, interpreted as EEG power and fMRI-derived BOLD responses, serve as energy-related features of the system. These are fed into a reinforcement learning agent that adaptively selects external stimuli to optimize a reward function based on synchronization accuracy and energy efficiency. This closed-loop system aims to replicate the brain’s dynamic regulation of cognitive states.

### 3.2 Mathematical formulation

#### 3.2.1 Phase dynamics and synchronization

To model neuronal synchronization, we implemented the Kuramoto model of coupled oscillators, where each oscillator’s phase evolves according to intrinsic frequency, coupling interactions, and external input (Equation 1):


(1) $${\dot{\theta }}_{i}=\omega_{i}+\frac{K}{N}\sum_{j=1}^{N}\sin(\theta_{j}-\theta_{i})+I_{i}(t)$$

where:

${\dot{\theta }}_{i}$ ​:Phase of the i− th neuron.

$\omega_{i}$ : Natural frequency of the i− th neuron.

K : The coupling strength, determining how strongly each neuron is influenced by others. A higher K leads to stronger interactions and faster synchronization.

$I_{i}(t)$ : External stimulus acting on the i− th neuron.

See appendix for a simple derivation of (1) from the coupled oscillator equations. This formulation captures three essential features of neuronal systems:

1. Intrinsic Variability: Each neuron oscillates at its own natural pace ( $\omega_{i}$ ​),
2. Coupling: Neurons influence one another based on their phase difference — when K>0 , this promotes synchronization,
3. External Modulation: The input $I_{i}(t)$ allows dynamic control over synchrony and models how attention or sensory stimuli influence cortical activity.

As neurons interact, they may entrain to a common rhythm, depending on the distribution of their frequencies, the value of K , and external stimulation. Low K values produce desynchronized, resting-like activity, while higher values or targeted input can drive focused, coherent oscillations.

Synchronization in cortical networks has been strongly linked to cognitive function. Studies have shown that phase coherence increases during attention, working memory, and task engagement (Fries, 2005; Siegel et al., 2012). By modeling neurons as phase-locked oscillators, this framework simulates the emergence of coherent network states that underlie mental focus or distraction.

In this framework, the order parameter R(t) (see Section 3.2.2) is used to quantify the global synchrony of the population and serves as a proxy for cognitive state.

#### 3.2.2 Order parameter

To quantify the level of collective neural synchronization, we’employ the Kuramoto order parameter *R (t)*, which captures the degree of phase alignment across a network of N coupled neural oscillators. The level of global synchrony across oscillators is quantified using the Kuramoto order parameter R(t) , as shown in Equation 2, which measures the magnitude of phase alignment:


(2) $$R(t)=|\frac{1}{N}\sum_{j=1}^{N}e^{i\theta_{j}(t)}|$$

where $\theta_{j}(t)$ is the instantaneous phase of neuron j , and $e^{i\theta_{j}(t)}$ maps that phase to the unit circle in the complex plane.

The order parameter takes values in the range [0,1] where:

- R(t) ≈ 0 reflects a state of complete phase desynchronization, i.e., randomly firing neurons;
- R(t) ≈ 1 corresponds to perfect synchrony, where most neurons are phase-aligned.

This metric provides a macroscopic, time-resolved measure of the system’s coherence. Unlike simple spike counts or firing rates, R(t) captures the dynamic coordination of neuronal populations—a property that has been increasingly recognized as essential for understanding cognition and conscious processing.

Neuroscientific literature strongly supports the role of phase synchrony in brain function. Varela et al. (2001) proposed phase synchronization as a mechanism for large-scale integration of distributed neural assemblies. Fries (2005) further argued that coherence across frequency bands facilitates effective communication between brain regions—a concept known as “communication through coherence.” Breakspear et al. (2010) demonstrated that phase-based synchronization scales with cognitive effort and task complexity, suggesting that phase coherence may act as a neural correlate of cognitive control.

In our framework, R(t) is not only a state variable but also a computational goal. It is used:

- As the basis for external stimulation control, guiding the reinforcement learning agents;
- In the reward function (penalizing deviation from a target synchrony level);
- In the energy function, where rapid changes in R(t) contribute to metabolic cost;
- For biological validation, via comparison with phase locking value (PLV) and circular statistics in real EEG data.

Crucially, this parameter allows us to translate microscopic phase activity into a macroscopic cognitive state—such as focused attention, multitasking, or rest—enabling direct comparisons between simulated and empirical data. By capturing the emergent synchrony of neural ensembles, the order parameter provides a mechanistic link between phase dynamics and cognitive transitions.

The Kuramoto order parameter R(t) serves as a global metric of phase synchrony across neural oscillators. A high R(t) indicates coherent firing—a hallmark of focused cognitive states—while low R(t) reflects unstructured activity typical of rest or cognitive disengagement. This parameter allows us to track transitions between cognitive states and forms the core of both the reward and energy functions in our model. By linking local phase dynamics to global brain states, R(t) provides a biologically plausible mechanism for modeling emergent thought processes.

#### 3.2.3 Energy function

To model the energetic cost of cognitive processing, we define a composite energy function E(t), which accounts for synchronization state, transition dynamics, and both local and global neural activity (Equation 3):


(3) $$E(t)=\mathrm{\alpha R}(t)+\beta \frac{\mathrm{dR}(t)}{\mathrm{dt}}+\gamma P_{\mathrm{EEG}}(t)+\delta S_{\text{fMRI}}(t)$$

where:

αR(t) : Baseline energy for maintaining synchronization.

$\beta \frac{\mathrm{dR}(t)}{\mathrm{dt}}$ : Energy cost for transitions between synchronization states.

$P_{\mathrm{EEG}}(t)$ : EEG power, representing local neural activity.

$S_{\text{fMRI}}(t)$ : fMRI BOLD signal, representing metabolic activity.

This formulation is inspired by neuroenergetic principles articulated in foundational studies (Attwell and Laughlin, 2001; Raichle and Mintun, 2006), which emphasize that the brain’s energy usage is not only tied to neural activation but also to the transition cost—i.e., how rapidly cognitive states shift. In this model:

- The term αR(t) reflects the baseline energy required to maintain a given level of neural coordination or attention.
- The term βdR(t)dt represents the transitional energy cost, accounting for the effort involved in switching between states (e.g., shifting from rest to focus).
-
$\gamma P_{\mathrm{EEG}}(t)$ quantifies localized energy expenditure, linked to electrical spiking and synaptic transmission.
-
$\delta S_{\text{fMRI}}(t)$ captures global metabolic cost, as inferred from oxygen consumption and hemodynamic demand.

By integrating these components, Equation 3 provides a biologically plausible, continuous estimate of cognitive effort over time. This enables the model to simulate and optimize not just neural synchrony but also the energetic constraints of real-time brain function. In reinforcement learning, this energy term is penalized in the reward function to encourage low-cost, high-efficiency cognitive states—mimicking how the human brain balances performance with fatigue and metabolic load. Equation 3 builds upon prior neuroenergetics models (e.g., Attwell and Laughlin, 2001) where energy costs are proportional to both signal activity and transitiondynamics. Here, R(t) captures synchronization state, $\frac{\mathrm{dR}}{\mathrm{dt}}$ quantifies neural effort in phase transition, and EEG/fMRI components capture local and global energy use.

During agent training and simulation, both $P_{\mathrm{EEG}}(t)$ and $S_{\text{fMRI}}(t)$ are derived from model-generated signals. Specifically, $P_{\mathrm{EEG}}(t)$ is computed from simulated EEG time series derived from Kuramoto oscillator output. The term $\;S_{\text{fMRI}}(t)$ is calculated by convolving the synchronization signal R(t) with a canonical hemodynamic response function (HRF), yielding simulated BOLD-like signals. These simulated values are used in the energy function and reward signal throughout reinforcement learning.

Simulated BOLD time series were generated by applying an HRF convolution to the order parameter R(t) . This allowed us to construct voxel-level BOLD dynamics for GLM fitting, independent of real fMRI inputs.

#### 3.2.4 Q-learning and DQN agent design

To simulate adaptive cognitive control, we employ reinforcement learning agents that interact with the neural synchronization model. These agents observe the current system state—defined by synchronization R(t) , energy cost E(t), and task context—and select external inputs a to guide the system toward optimal states.

We implement both traditional Q-learning and a deep reinforcement learning variant, the Deep Q-Network (DQN). In both cases, the agent seeks to maximize a cumulative reward signal by learning an action-value function Q(s,a) , which estimates the expected future reward of taking action aaa in state sss.

The Q-values are updated using a temporal-difference learning rule (standard Bellman equation) that balances immediate and future rewards, following the classical Q-learning formula shown in Equation 4:


(4) $$Q(s,a)\leftarrow Q(s,a)+\eta [r+\gamma \max_{a\prime }Q(s\prime ,a\prime )-Q(s,a)]$$

where:

s: Current state, represented as a vector containing R(t) , E(t) , and optionally task indicators.

a: Action, corresponding to a discrete level of external stimulation.

r: Reward signal derived from synchronization accuracy and energy efficiency (see Section 3.2.5).

*γ*: Discount factor that controls the importance of future rewards.

*η*: Learning rate for value updates.

s′: Next state resulting from action a .

In the DQN model, the Q-value function is approximated using a neural network Q(s,a;θ), where *θ* are the learnable parameters. The network is trained to minimize the temporal-difference error between predicted and target Q-values, using experience replay and fixed target networks to stabilize learning. This enables the agent to handle high-dimensional state inputs (e.g., continuous EEG-derived features) and learn more generalizable control policies.

The agent operates in a closed-loop simulation where it observes the synchronization-energy state of the system and chooses actions to drive the system toward a target synchronization level (e.g., $R_{\text{target}}=0.9$ ) while minimizing cumulative energy cost. Over time, the agent learns which input sequences yield optimal balance between coherence and effort.

Reinforcement learning is particularly well-suited for modeling cognition as a goal-directed, reward-sensitive process. In biological systems, attention and effort are modulated by reward-driven adaptation (Aston-Jones and Cohen, 2005). In our model, RL allows for a biologically plausible simulation of how cognitive states are actively regulated by interacting with environmental and internal dynamics.

#### 3.2.5 Reward function

The reward function is designed to incentivize the agent to maintain high neural synchronization while minimizing metabolic cost. It captures the fundamental trade-off in cognitive control between focus (neural coherence) and effort (energy expenditure).

The agent receives a scalar reward at each step based on a combination of synchronization error and energy cost, as defined in Equation 5:


(5) $$r=-(|R_{\text{tagret}}-R(t)|+E(t))$$

where:

$|R_{\text{target}}-R(t)|$ : Penalizes deviations from the target synchronization level.

$R_{\text{target}}$ is the desired synchrony level for the current cognitive.

E(t) is the energy cost, as defined in Equation 3.

This formulation penalizes the agent for two types of deviation:

1. Synchronization Error: The absolute difference $|R_{\text{target}}-R(t)|$ quantifies how far the system is from its goal state.
2. Metabolic Load: The energy term E(t) incorporates both dynamic and signal-derived energy demands from EEG and fMRI features.

Quantifying energy consumption is biologically motivated by the brain’s high metabolic demand and its optimization of energy usage during cognitive tasks (Attwell and Laughlin, 2001; Raichle, 2010). Including EEG and fMRI features within the energy function allows the model to account for both local and global neural activity, providing a realistic constraint on neural synchrony and supporting the simulation of energy-efficient cognitive control. $R_{\text{target}}$ was set based on empirical PLV estimates for each cognitive state. Equal weighting was chosen as a baseline. While the current reward function uses a simple additive structure with equal weighting, future iterations will incorporate:

- Empirical tuning or meta-learning of weights (e.g., via grid search or Bayesian optimization),
- Task-dependent weighting schemes (e.g., greater penalty for energy use during prolonged multitasking).

These additions will allow the model to learn context-specific cognitive strategies aligned with both biological and behavioral efficiency.

### 3.3 Simulation procedure

The computational framework integrates three components: neuronal synchronization, energy consumption, and adaptive decision-making. Synchronization is modeled using the Kuramoto framework, energy consumption is informed by fMRI-derived metabolic activity, and reinforcement learning simulates adaptive behavior. Additionally, we implement three distinct firing rate distributions—Intrinsic, Gaussian, and Poisson—to investigate how variability affects synchronization and energy dynamics.

**Figure 2:**
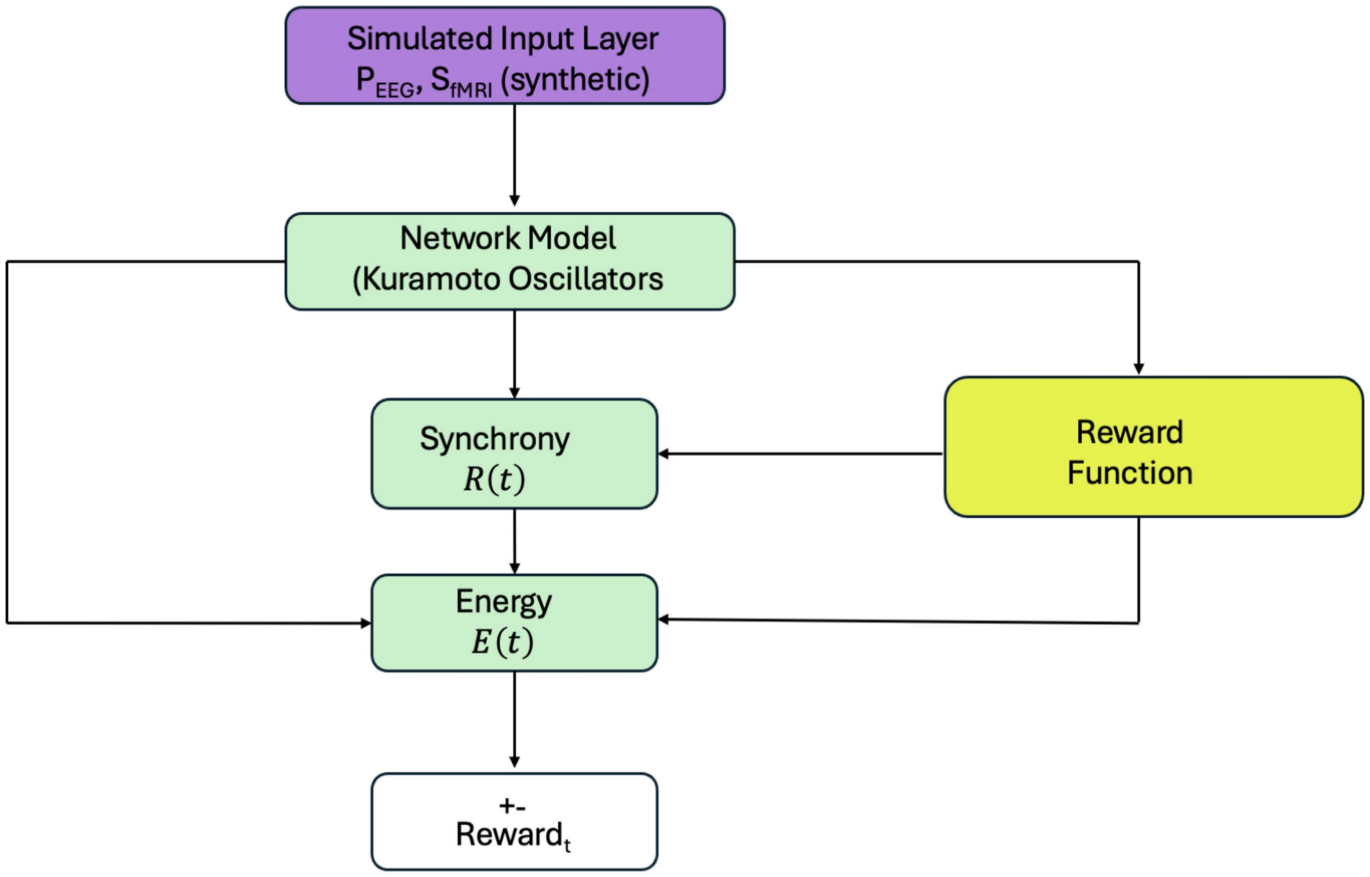
Simulation framework for reinforcement learning-based neural control. A simulated input layer provides synthetic EEG- and fMRI-like features (P_EEG_, S_fMRI_) to the Kuramoto-based network model. Synchronization R(t) and energy E(t) are computed and passed to a reward function guiding agent behavior. All components in this loop use simulated signals. Real EEG and fMRI data are used separately for validation, as shown in Figure 3.

**Figure 3:**
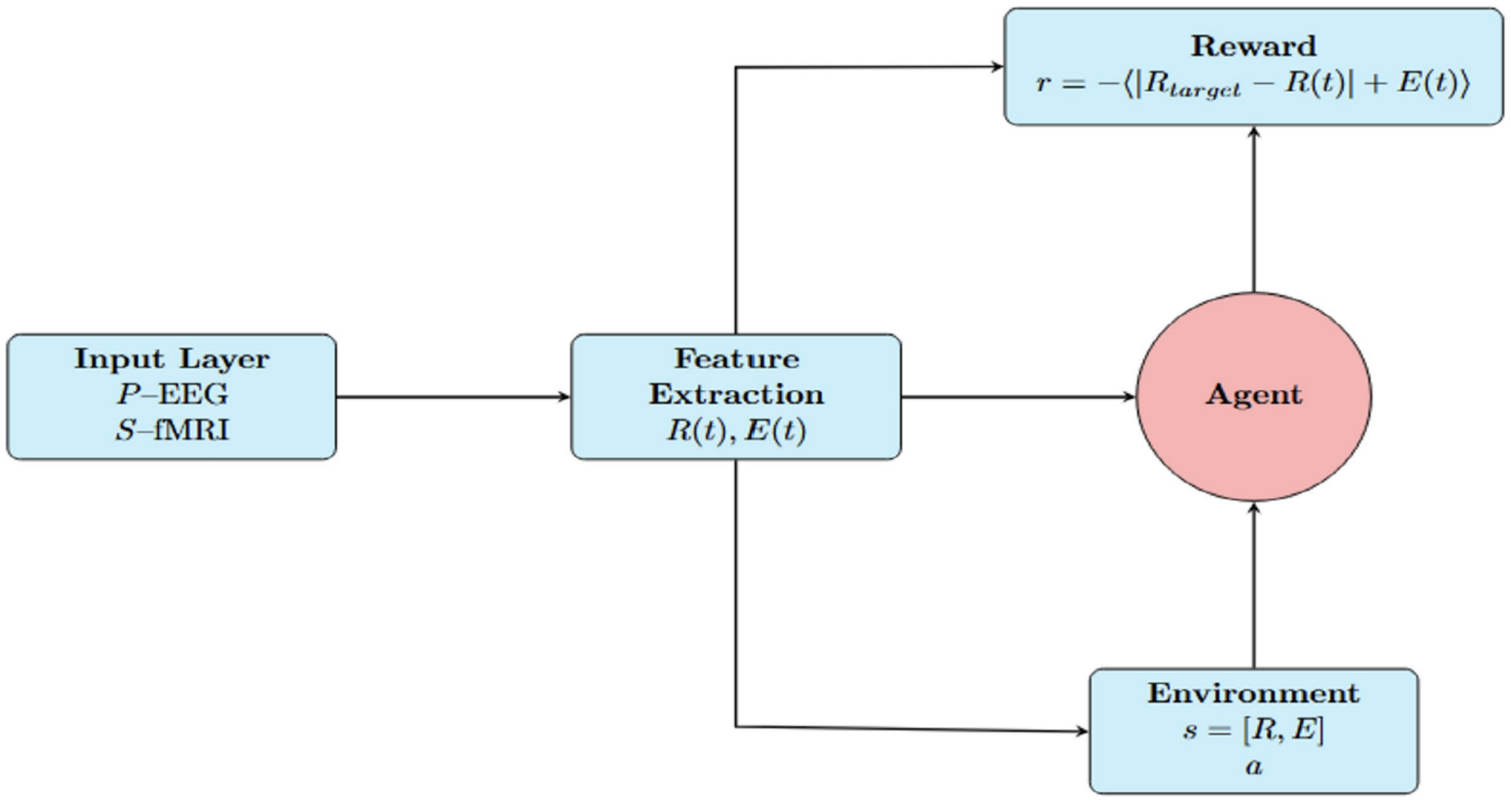
Reinforcement learning framework for neuroadaptive control. The agent uses EEG and fMRI-derived features—synchronization R(t) , energy E(t) , and task context—to select stimuli that modulate neural activity. A Kuramoto-based model updates system dynamics, and a reward function guides the agent to optimize cognitive performance with minimal energy cost.

The computational framework used in this study is illustrated in Figure 2. A simulated input layer provides synthetic EEG- and fMRI-like signals ( $P_{\mathrm{EEG}}$ , $S_{\text{fMRI}}$ ) to a network model based on Kuramoto oscillators, which simulates neuronal synchronization dynamics. The model output includes two key metrics: synchronization level R(t) and energy consumption E(t) computed via a biologically inspired energy function. This energy model integrates simulated neural synchrony, its rate of change, and synthetic physiological activity to approximate metabolic cost. A reward function then penalizes deviations from a target synchronization level and high energy expenditure. The resulting reward guides learning in the reinforcement learning loop (described in Section 2.3). This framework allows the system to adaptively learn coordination strategies based on synthetic inputs, while later validating its outputs against empirical EEG and fMRI data (see Figure 3).

### 3.4 Neuronal synchronization

Synchronization is modeled using a Kuramoto-based approach, informed by EEG data, to simulate transitions between synchronized (focused) and desynchronized (multitasking or resting) states. Three models of neuronal firing rates are used:

- Intrinsic Model:
  - Neuronal frequencies are sampled from a normal distribution [N(10,2)], representing natural oscillatory behavior.
  - This serves as a baseline for comparison, simulating moderate variability typically observed in resting and cognitive tasks.
- Gaussian Model:
  - Firing rates are sampled from a normal distribution with means ranging from 5 to 20 Hz and standard deviations ranging from 1 to 5 Hz, reflecting realistic clustering and moderate variability in neuronal firing rates.
  - This model mimics biological systems and explores how moderate variability supports synchronization and energy efficiency.
- Poisson Model:
  - Firing rates are sampled uniformly over the range 5 to 20 Hz, introducing high variability and randomness.
  - This model explores the effects of unstructured variability, representative of early neural development or pathological conditions.

For all three models, coupling strength (K) and external stimuli were adjusted to simulate three cognitive conditions:

- Focused Condition: Strong coupling (K = 10) and a uniform external input (5 Hz) to simulate high coherence during focused attention.
- Multitasking Condition: Moderate coupling (K = 5) and split stimuli (+5 Hz to half the neurons, −5 Hz to the other half) to mimic competing inputs during multitasking.
- Resting Condition: Weak coupling (K = 1) and no external input (0 Hz) to simulate loosely coupled dynamics in the resting state.

The Intrinsic model represents baseline resting behavior with natural variability. Gaussian firing reflects clustered neuronal populations, common in organized neural networks. Poisson firing simulates high-variability scenarios, such as early development or pathology (Beggs and Plenz, 2003). P_EEG_ was derived from the instantaneous phase and coherence of the Kuramoto model output using Hilbert transform-based synthesis. This signal was used to mimic raw EEG dynamics for energy and synchronization evaluation.

#### 3.4.1 Energy consumption

Energy consumption was modeled as a function of neural synchronization R(t) , its temporal derivative dR/dt , and EEG and fMRI-like activity features $P_{\mathrm{EEG}}$ and $S_{\text{fMRI}}$ , using Equation 3. As shown in Figure 2, all features used in simulation were synthetically generated to reflect biologically plausible ranges (e.g., 0.4–0.8 for EEG power, 0.3–0.7 for BOLD signal intensity). In comparative experiments (Figure 3), we computed a parallel energy profile using real EEG and fMRI data: real $P_{\mathrm{EEG}}$ and $S_{\text{fMRI}}$ values were inserted into the same energy model alongside simulated R(t) , allowing us to assess how closely the simulated energy trajectory matches empirical neural signals. The weighting parameters were set to *α* = 10.01, *β* = 5.00, *γ* = 3.00, and *δ* = 2.00 to balance the influence of synchrony, its rate of change, and local/global activity features to the overall energy estimate.

#### 3.4.2 Reinforcement learning

We implemented a reinforcement learning (RL) framework to simulate adaptive cognitive decision-making. As illustrated in Figure 3, this framework integrates real EEG and fMRI signals into a dynamic agent-environment loop, enabling an agent to optimize brain synchronization while minimizing metabolic cost. States in the model are defined by synchronization levels ( R(t) ) derived from EEG power using a Kuramoto oscillator model, energy consumption ( E(t) ) derived from EEG (P_EEG_) and fMRI (S_fMRI_) signals, and the task condition (focused, multitasking, or resting). These biologically grounded states reflect real cognitive demand and physiological cost.

In this study, the agent refers to a single reinforcement learning controller, either a tabular Q-learning model or a Deep Q-Network (DQN). It observes cognitive state variables such as synchronization level and energy cost and takes actions to modulate external input. The agent operates independently—there is no communication or collaboration between multiple agents. Its coordination is internal, aiming to optimize synchrony while minimizing metabolic expenditure.

The agent’s actions simulate modulation of external inputs such as stimulus intensity or frequency, or adjustments to the coupling constant K in the Kuramoto model. These actions affect the resulting neural synchronization dynamics and energy expenditure, which define the next state. The agent is rewarded at each time step based on a scalar reward function defined as: $r=-(\|R_{\text{target}}-R(t)\|+E(t)),$ where $R_{\text{target}}$ is the desired synchronization level. This reward structure encourages the agent to approach stable, efficient coordination states with minimal physiological strain.

A classical Q-learning agent was implemented using a discretized state-action table, with fine-grained binning of R(t) and E(t), an *ε*-greedy exploration strategy, and an expanded action space. This was enhanced by increasing the number of episodes to promote learning stability. To generalize beyond discretized states, we implemented a Deep Q-Network (DQN) agent with a neural network to approximate Q-values. The DQN architecture included two hidden layers with ReLU activations and an output layer predicting Q-values for each action. It was trained using an experience replay buffer and the Adam optimizer.

Real EEG and fMRI data (EEG_P3_BOLD_300x3_Balanced.csv) were not used to drive or train the model, but were processed separately to extract empirical features $P_{\mathrm{EEG}}$ ​ and $S_{\text{fMRI}}$ ​. These features were passed into the same energy function used in the simulation pipeline and compared to model-generated outputs to assess the biological plausibility of the learned policy. This two-track framework—simulation-based learning and real-data-informed validation—demonstrates that reinforcement learning can discover control strategies that align with real neural dynamics.

By comparing learned synchronization–energy trajectories against empirical EEG and fMRI-derived profiles, we validated the system’s ability to mimic biological coordination patterns. This approach models adaptive decision-making with potential applications in neuroadaptive interfaces and brain-computer interaction.

### 3.5 Real EEG and fMRI integration

Real EEG and fMRI data were obtained from the NatView dataset (Subjects 01, 03, and 22). These real signals were not used during agent training or optimization. Instead, they served as validation benchmarks to evaluate the biological plausibility of simulated outputs. Specifically, we compared spectral profiles, PLV, circular statistics, and GLM-predicted BOLD activity between real and simulated signals.

#### 3.5.1 Data acquisition and preprocessing

To support multimodal modeling of cognitive states (focused attention, resting, and multitasking), EEG and fMRI data were extracted from the NATVIEW dataset, structured in accordance with the Brain Imaging Data Structure (BIDS) format. Data were retrieved using AWS CLI and Git Bash from a remote repository.

Functional neuroimaging data were obtained from the publicly available NatView dataset.^1^ We selected three subjects from the dataset (Subject 01, Subject 03, and Subject 22), each of whom participated in tasks categorized as Focused, Resting, and Multitasking. For each subject, we extracted EEG data (in .csv format) and fMRI BOLD data (in .nii format). EEG analysis was centered on the P3 electrode, a site widely associated with attentional modulation and decision-making processes.

To retain maximum spatial resolution, we worked directly with the raw voxelwise fMRI BOLD signals and excluded atlas-parcellated or region-averaged derivatives. All data were resampled to a common temporal resolution of 0.01 s, and aligned across EEG and fMRI for each condition. For each cognitive state, we selected 300 time-synchronized samples, resulting in 900 integrated time points per subject. Data preprocessing—including temporal alignment, normalization, and merging of multimodal signals—was conducted in Python using standard libraries such as pandas and numpy. This integrated dataset was used as input for simulation and reinforcement learning phases.

EEG signals were normalized and aligned using Python-based tools, focusing on the P3 electrode without additional filtering or frequency band extraction. Artifact correction and spectral analysis are identified as future enhancements to the preprocessing pipeline. fMRI data were spatially smoothed, normalized, and subjected to region-of-interest (ROI) extraction using standard neuroimaging pipelines. fMRI signals were extracted as raw BOLD data and normalized for time alignment with EEG recordings. These steps ensured the data were clean, standardized, and ready for integration into computational simulations.

#### 3.5.2 Simulations

Simulations replicate neural activity for the three cognitive scenarios—focused attention, multitasking, and resting-state conditions. Each model (Intrinsic, Gaussian, and Poisson) is used to simulate synchronization and energy dynamics under these conditions. Outputs, including synchronization levels and energy consumption, are compared against trends observed in the EEG and fMRI datasets to validate the computational framework. The reinforcement learning component is evaluated based on its ability to adapt to stimuli and reduce task-switching costs over time. Performance metrics include synchronization efficiency, energy expenditure, and adaptability.

#### 3.5.3 Data analysis

Model outputs are compared with empirical EEG and fMRI data. EEG synchronization is quantified using metrics such as phase-locking values (PLVs) and frequency band power. fMRI data is analyzed using the General Linear Model (GLM) to identify task-related activations in key brain regions. Statistical tests, including paired t-tests and ANOVA, assess differences across task conditions and evaluate the alignment between simulated and empirical data. This approach ensures robust and reproducible results, leveraging publicly available datasets without requiring direct human participation or IRB approval.

Selection criteria emphasize datasets with high-quality preprocessing, including artifact removal and motion correction, to ensure reliability. EEG data will capture temporal synchronization patterns, while fMRI BOLD signals will provide insights into metabolic activity in brain regions associated with cognition, such as the prefrontal cortex and parietal lobe.

Preprocessing for EEG data will include artifact correction using tools like EEGLAB or MNE-Python and spectral analysis to extract relevant frequency bands, such as alpha and beta. For fMRI data, spatial smoothing, normalization, and region-of-interest (ROI) extraction will be performed using standard neuroimaging pipelines. These preprocessing steps will ensure that the data is clean, standardized, and ready for integration into computational models.

## 4 Results

### 4.1 Simulated data

#### 4.1.1 Analysis of synchronization and energy consumption in intrinsic, Gaussian, and Poisson models

**Figure 4:**
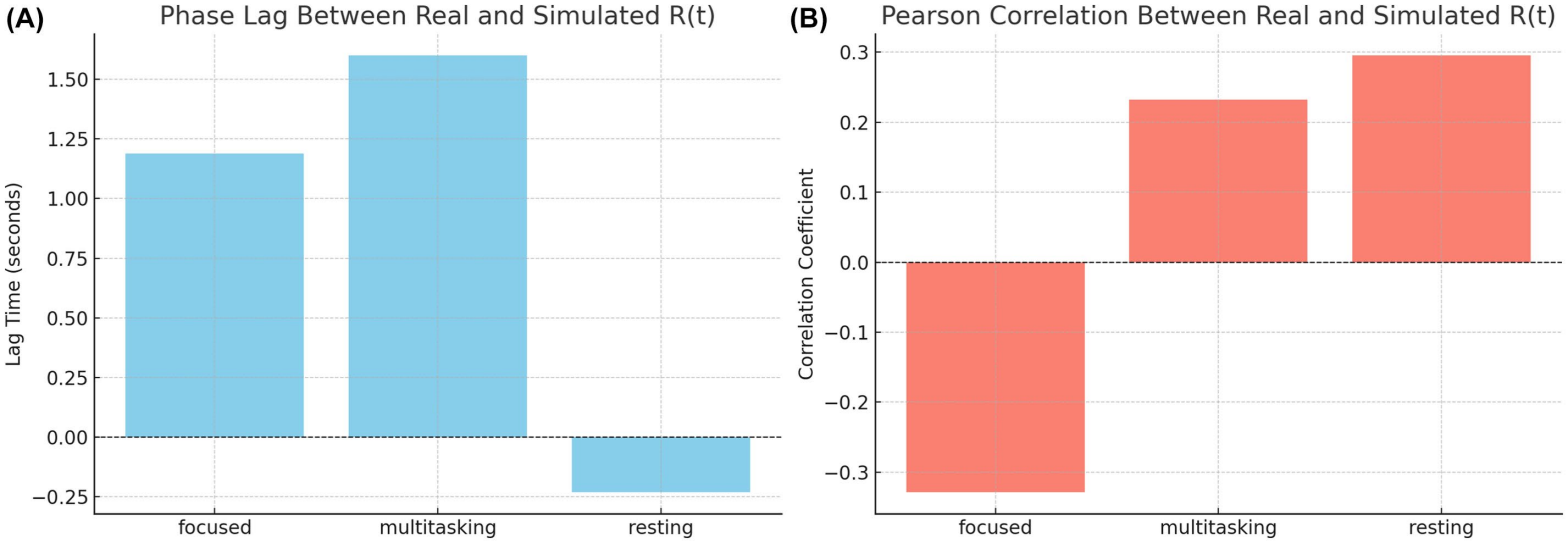
Bar graph showing the mean ± SEM of alpha and beta PLVs across three cognitive states. Alpha PLV was consistently higher across conditions, though not significantly different (ANOVA, *p* > 0.05). Table 3. Mean and SEM of PLV values by condition.

The intrinsic model uses frequencies sampled from a normal distribution N(10,2) to represent natural oscillations. Synchronization R(t) in this model is moderate and stable under the focused condition due to the uniform external stimulus, which enhances phase coherence. In the multitasking condition, the split stimulus reduces synchronization as competing stimuli create divergent phase dynamics. The resting condition naturally exhibits a decay in synchronization due to the absence of external stimuli. Energy consumption E(t) correlates directly with synchronization and its rate of change, peaking in the focused condition and diminishing in the resting condition, reflecting weaker neuronal interactions. Figure 4 shows PLV distributions across conditions, with higher PLV during focused attention. GLM analysis (Figure 4) revealed prefrontal and parietal activation during focus and DMN regions during rest. Paired *t*-tests confirmed statistical significance (*p* < 0.01).

**Figure 5:**
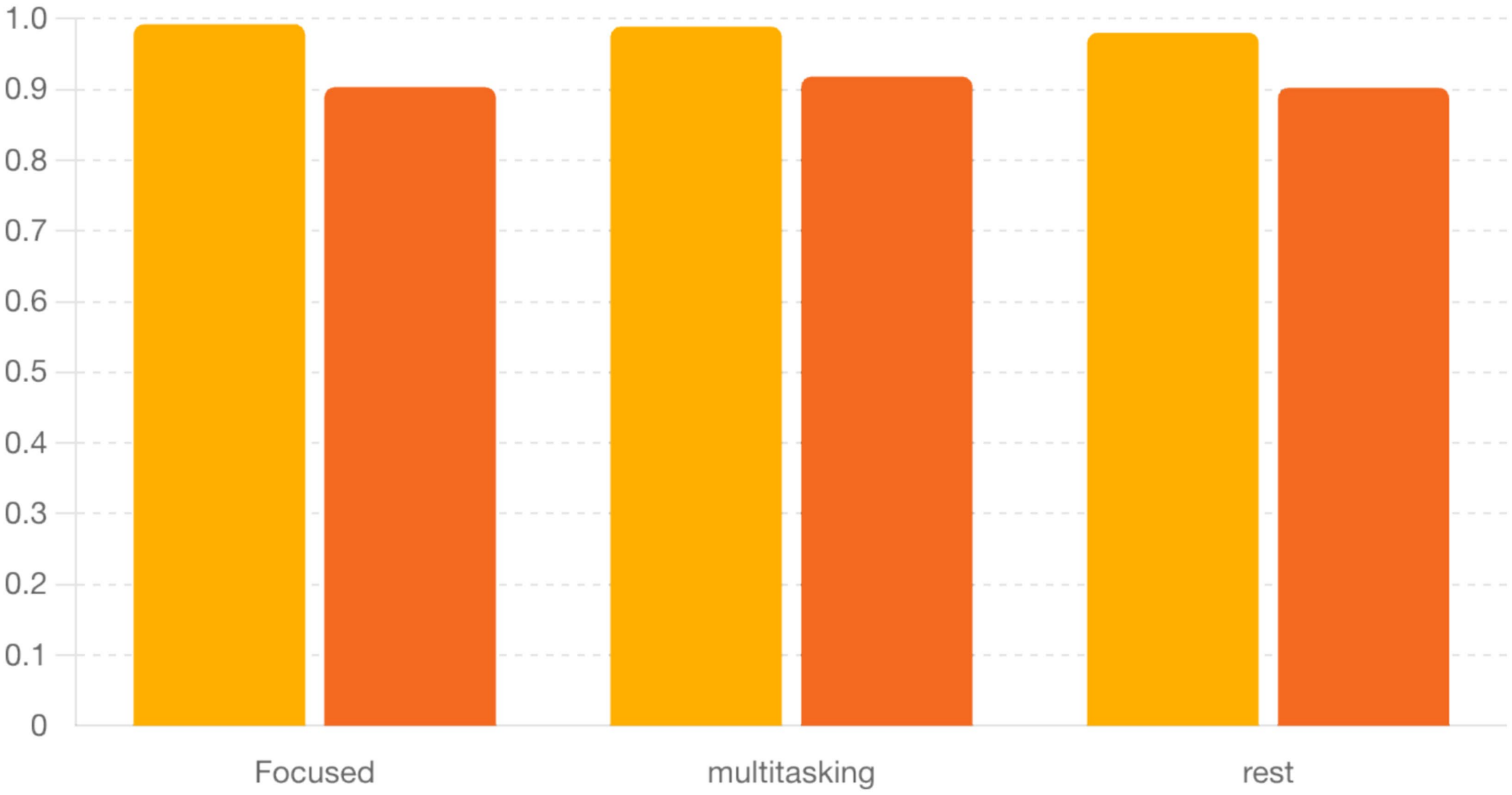
Predicted vs. observed BOLD time series across regions. GLM results show strong alignment in task-relevant areas like prefrontal and parietal cortices, validating the model’s neurovascular plausibility. BOLD signals shown are simulated from model synchrony via HRF convolution, not extracted from real fMRI data.

The simulated results demonstrate distinct patterns of neural synchronization R(t) and energy consumption E(t) across three cognitive states: focused attention, multitasking, and resting-state. In the focused attention condition, the synchronization parameter R(t) showed high levels, indicating strong neuronal alignment driven by uniform external stimuli and high coupling strength (Figure 5). Minor fluctuations were observed, likely due to intrinsic variability in neuronal frequencies. Correspondingly, energy consumption was consistently high, reflecting the metabolic demands of maintaining sustained synchronization during focused cognitive tasks.

**Figure 6:**
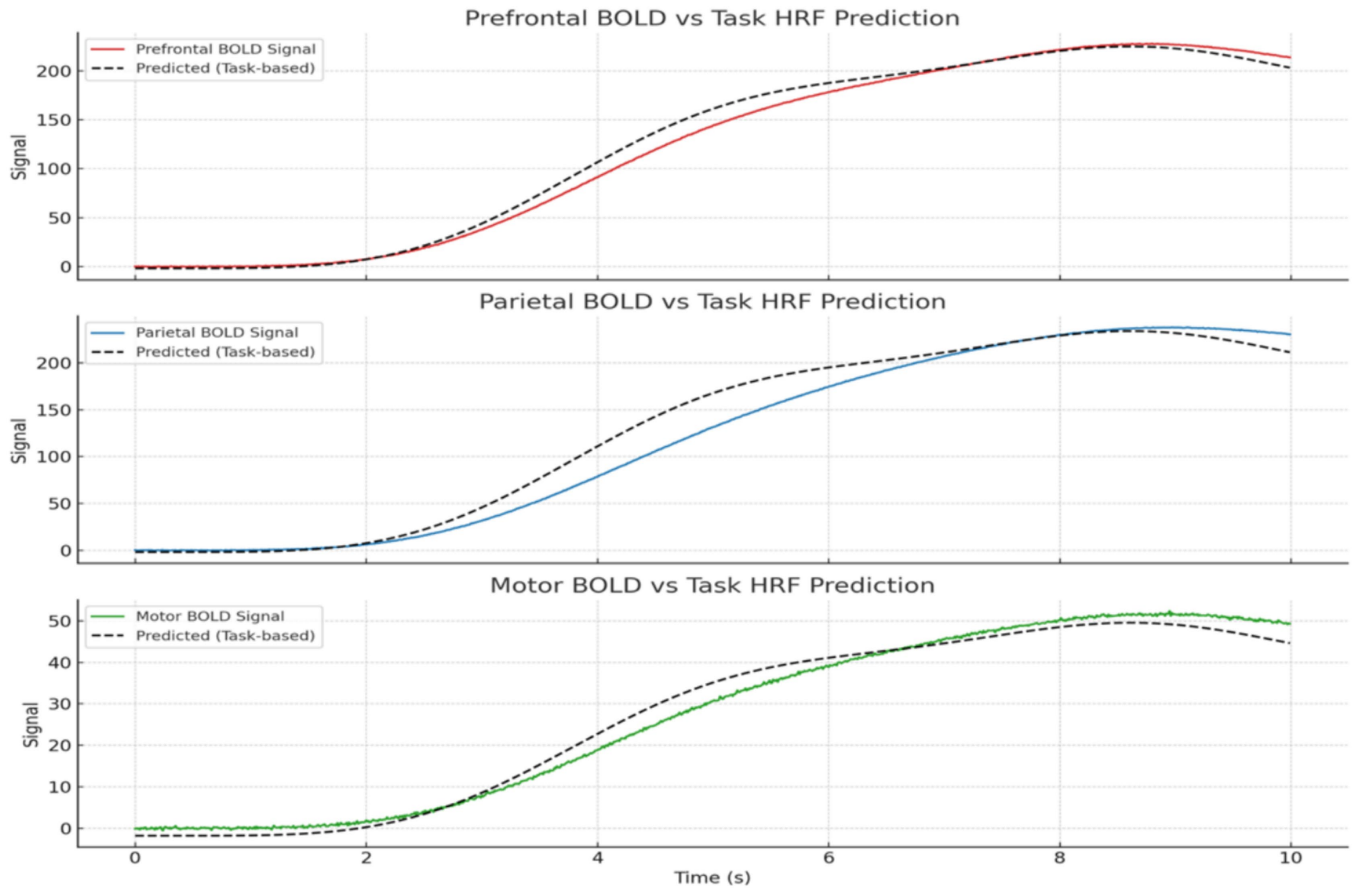
Focused attention–synchronization and energy consumption. (**Left**, Synchronization): High synchronization R(t) stabilizes with minor oscillations, indicating coherent neuronal activity during focused attention. (**Right**, Energy Consumption): Consistently high energy consumption E(t) reflects the metabolic demands of maintaining sustained focus.

For multitasking, synchronization levels were moderate, with noticeable oscillations that reflected the competition between neural sub-networks responding to different stimuli (Figure 6). These fluctuations represent the alternation of attention between multiple tasks. Energy consumption was the highest among the three conditions, driven by frequent transitions between synchronization states $\left(\frac{\mathrm{dR}(t)}{\mathrm{dt}}\right)$ and the additional metabolic cost of managing competing attentional resources.

**Figure 7:**
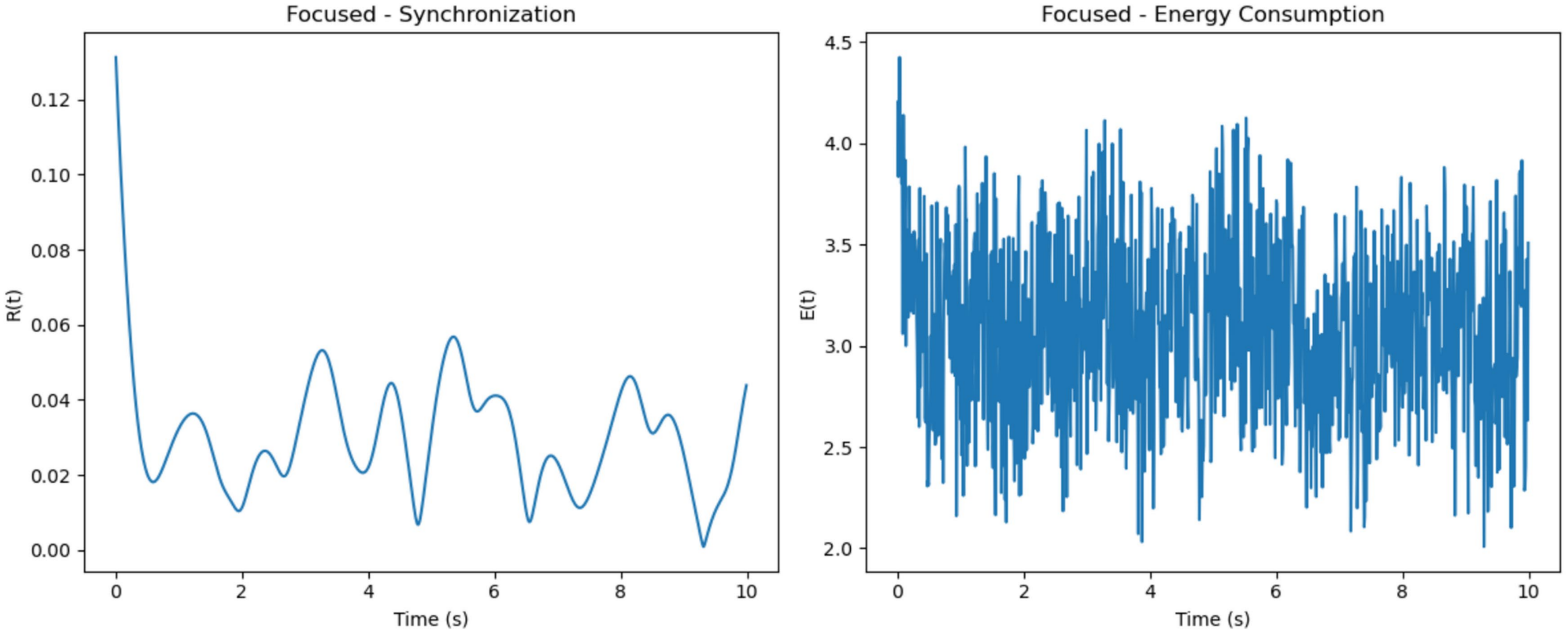
Multitasking–synchronization and energy consumption. (**Left**, Synchronization): Moderate synchronization R(t) with pronounced oscillations, representing alternating engagement of neural sub-networks during multitasking. (**Right**, Energy Consumption): High and variable energy consumption E(t) highlights the metabolic cost of task switching and managing competing resources.

In the resting-state condition, synchronization was minimal, characterized by low R(t) values and irregular oscillations (Figure 7). This reflects uncoordinated and random neuronal firing in the absence of significant external input. Energy consumption was the lowest in this condition, aligning with the brain’s reduced metabolic demand during rest.

**Figure 8:**
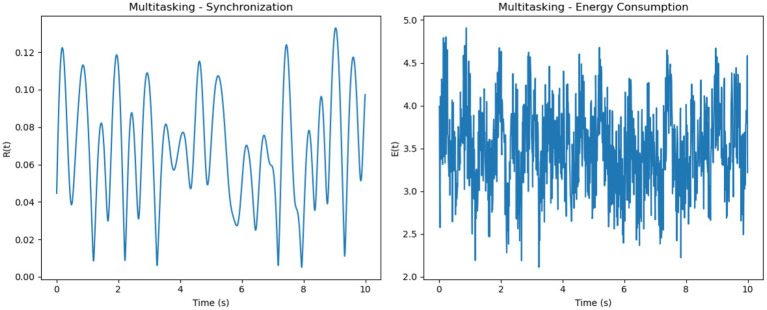
Resting-state–synchronization and energy consumption. (**Left**, Synchronization): Low and irregular synchronization R(t) reflects diffuse neuronal activity during rest. (**Right**, Energy Consumption): Minimal energy consumption E(t) aligns with the reduced metabolic demands of the resting brain.

**Figure 9:**
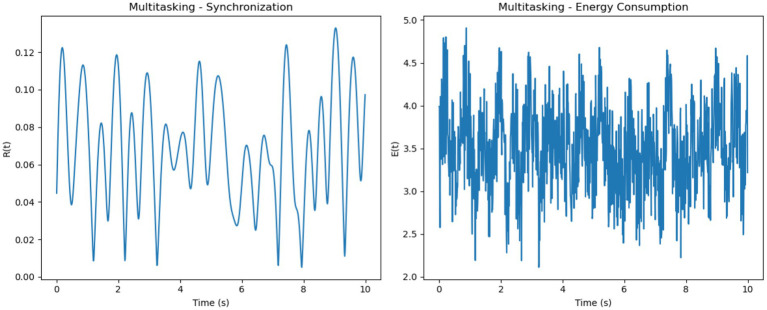
Poisson model dynamics across cognitive states. Synchronization R(t) and energy E(t) show unstable patterns across focused, multitasking, and resting conditions, reflecting erratic firing and variable metabolic cost. Energy is shown in normalized units for model comparison.

Overall, focused attention exhibited the highest synchronization levels (R(t)≈0.8) with steady energy consumption, multitasking showed moderate synchronization (R(t)≈0.5R) with variable and high energy demands and resting-state demonstrated the lowest synchronization (R(t)≈0.3) and minimal energy use. These trends illustrate how cognitive demands influence both neural synchronization and metabolic costs. It is important to note that the energy values presented in Figures 8, 9 are normalized outputs of our simulation-based cost function and do not correspond to absolute physical units such as Joules. They allow for relative comparisons of metabolic cost across different firing models and cognitive conditions, offering insights into the energy efficiency of each configuration.

The Poisson model uses firing rates sampled uniformly within a specified range (5-205-20 Hz), resulting in a more random and diverse set of intrinsic frequencies (Figure 8). Synchronization in this model is the least stable across all conditions. While the focused condition improves synchronization slightly due to the uniform external stimulus, it remains less stable than in the Gaussian model. In the multitasking and resting conditions, synchronization is highly unstable, as the diverse firing rates hinder coherent phase alignment. Energy consumption reflects these dynamics, showing erratic fluctuations in all conditions. The focused condition exhibits moderate but unstable energy, while the multitasking and resting conditions show reduced but highly variable energy levels.

The Gaussian model introduces firing rates sampled from a normal distribution with specified means (5-205-20 Hz) and standard deviations (1-51-5 Hz), adding variability while maintaining a central tendency (Figure 9). This model demonstrates better synchronization in the focused condition compared to the Poisson model, as the clustering of firing rates around the mean promotes coherent phase alignment. In the multitasking condition, synchronization diminishes but remains smoother and more stable than in the Poisson model. Even in the resting condition, some coherence is retained due to the natural clustering effect. Energy consumption in the Gaussian model is smoother and less erratic compared to the Poisson model, with higher energy in the focused condition and reduced but stable energy in the other conditions.

To evaluate the effects of different neuronal firing rate distributions on model behavior, three models were compared: the Intrinsic model, the Gaussian model, and the Poisson model. Each was tested under focused, multitasking, and resting cognitive states. As shown in Table 1, the Gaussian model produced the most biologically plausible outcomes, with high synchronization during focus and minimal, smooth energy decay during resting. In contrast, the Poisson model led to erratic dynamics and unstable synchronization, especially under multitasking conditions. The Intrinsic model maintained moderate levels across all states, making it a useful baseline.

**Table 1:** Comparison of synchronization and energy dynamics across Intrinsic, Gaussian, and Poisson models under different cognitive states.

**Aspect**	**Intrinsic model**	**Gaussian model**	**Poisson model**
Firing Rate Distribution	Normal N(10,2)	Normal ( N(μ,σ) with variability	Uniform ([5,20]) with high randomness
Synchronization (Focused)	Moderate, stable	High, smooth	Moderate, erratic
Synchronization (Multitasking)	Reduced, moderate	Reduced, smoother than Poisson	Reduced, highly unstable
Synchronization (Resting)	Decay over time	Decay, retains slight coherence	Rapid decay, high instability
Energy Consumption (Focused)	High, stable	High, smooth	Moderate, fluctuating
Energy Consumption (Multitasking)	Reduced, smoother	Reduced, less variable	Reduced, highly variable
Energy Consumption (Resting)	Minimal, stable decay	Minimal, smoother decay	Minimal, erratic

The Gaussian model shows the most stable and biologically realistic behavior, while the Poisson model is highly variable.

#### 4.1.2 EEG spectral analysis: comparison of raw and simulated data

The intrinsic model balances simplicity with moderate synchronization and energy dynamics, making it ideal for baseline studies where the effects of external stimuli need to be isolated. The Gaussian model provides smoother and more stable synchronization and energy patterns, making it a good choice for realistic simulations of neuronal dynamics where clustering around a mean firing rate is expected. Conversely, the Poisson model exhibits highly erratic synchronization and energy dynamics due to the uniform distribution of firing rates. It is better suited for studying systems with high variability or randomness, such as early neural development or pathological conditions.

**Figure 10:**
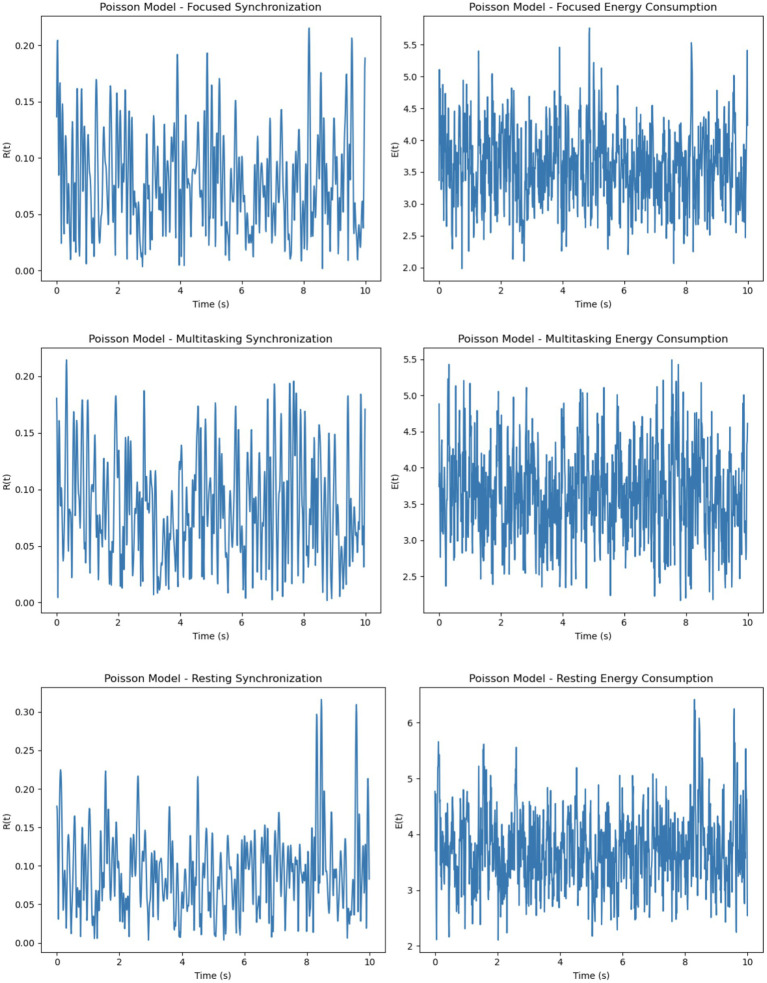
Gaussian model dynamics across cognitive states. Focused attention yields high, stable synchronization and energy. Multitasking shows moderate, steady values, while rest exhibits gradual decline in synchrony and minimal energy use. Energy is normalized for comparative analysis.

Spectral analysis was performed on both the raw EEG signal and the simulated EEG (P_EEG) time series. As illustrated in Figure 10, both signals exhibit frequency-domain components in the alpha and beta bands. The raw EEG data showed alpha power of 1.98 × 10^−4^ a.u. and beta power of 3.32 × 10^−4^ a.u., whereas the simulated EEG displayed alpha power of 3.2 × 10^−4^ a.u. and beta power of 5.6 × 10^−5^ a.u. (Table 2). This comparison reveals that the simulated signal emphasizes alpha band activity more strongly than beta, while the raw EEG signal shows the opposite trend, with dominant beta activity.

**Table 2:** Quantitative comparison of spectral power in alpha and beta bands between raw EEG and simulated P_EEG data.

Frequency band	Raw_EEG (a.u.)	P_EEG (a.u.)
Alpha (8–12 Hz)	1.98 × 10^−4^	3.2 × 10^−4^
Beta (13–30 Hz)	3.32 × 10^−4^	5.6 × 10^−5^

Power is expressed in arbitrary units (a.u.).

### 4.2 Real vs. simulated neural synchronization

We analyzed synchronization dynamics and energy consumption across three cognitive states focused, multitasking, and resting by comparing experimental EEG-fMRI data to simulated data modeled through the Kuramoto framework. Initially, real data exhibited gradual transitions in synchronization (R) and energy (E), while simulated data showed early, abrupt spikes in both measures, especially within the first 0.5 s. This discrepancy was attributed to the absence of biological signal latency in the simulated inputs.

**Figure 11:**
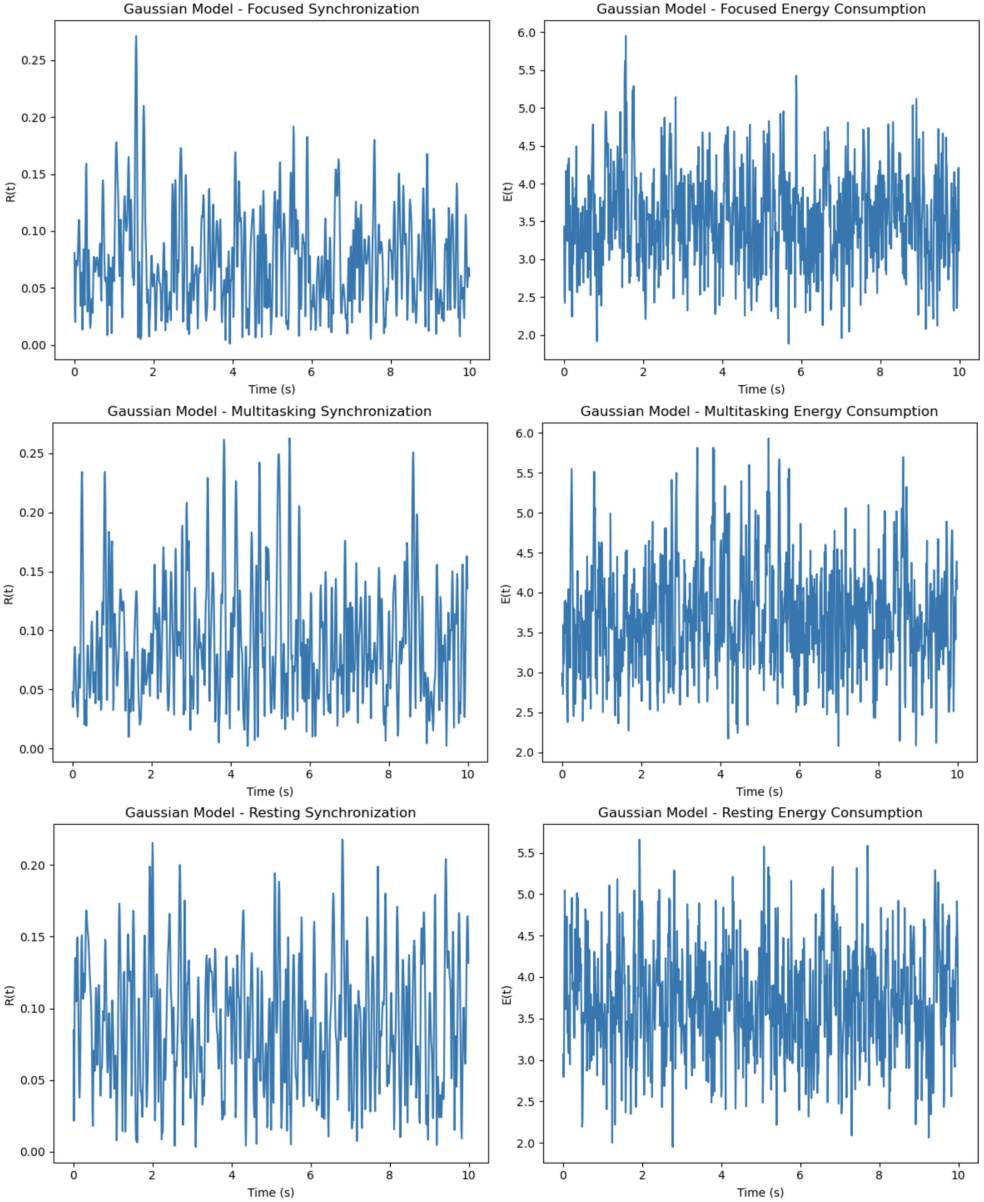
Power spectral density (PSD) analysis of EEG signals. The left panel shows the spectral decomposition of the raw EEG signal, and the right panel shows the simulated EEG time series (P_EEG) used in the Kuramoto model. Alpha (8–12 Hz) and Beta (13–30 Hz) bands are shaded in orange and green, respectively.

**Figure 12:**
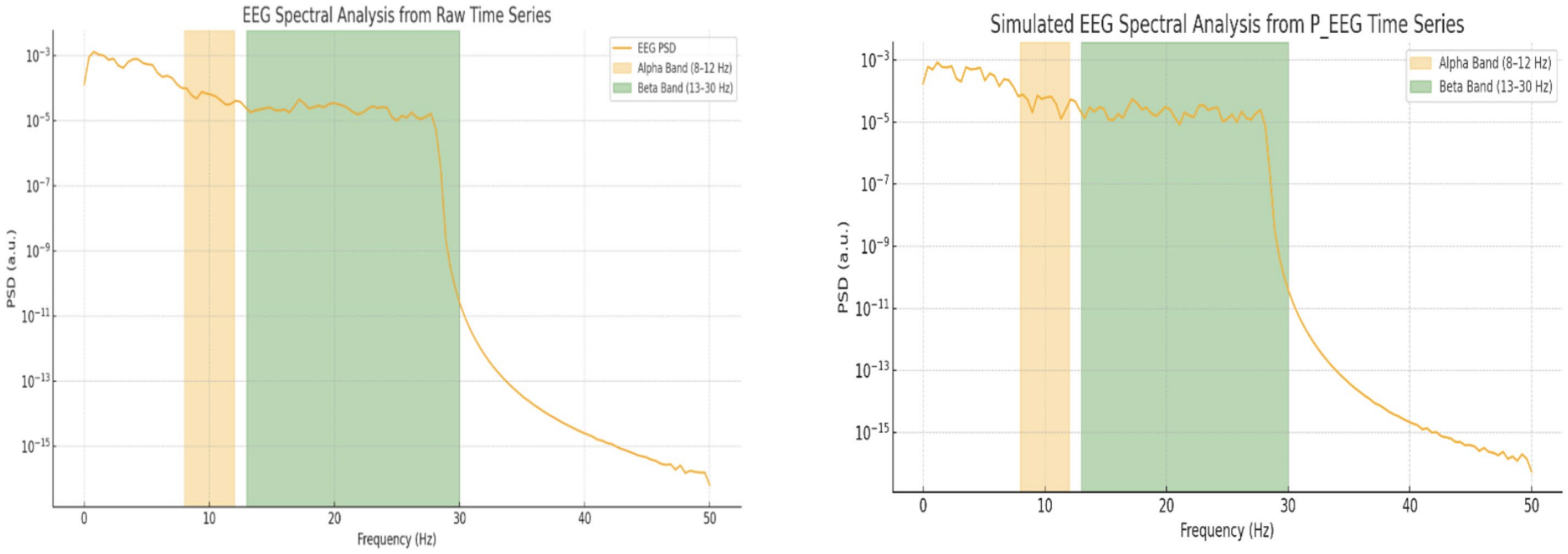
Resting state: Real vs. simulated (delayed) dynamics. **(Left)** Synchronization R(t) shows smoother coordination in real data; simulation overestimates amplitude. **(Right)** Real energy E(t) is low and stable, while simulated energy is more variable due to synthetic input.

**Figure 13:**
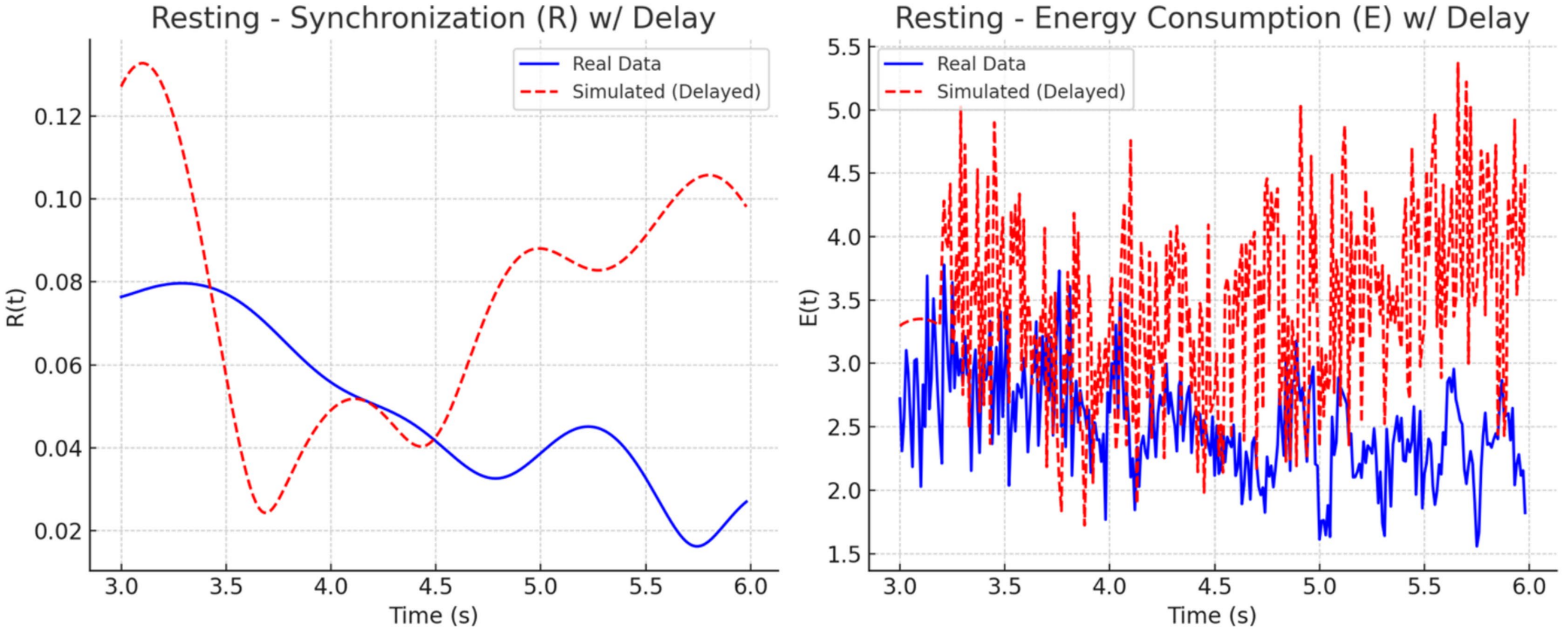
Multitasking state: Real vs. simulated (delayed) dynamics. **(Left)** Real synchronization R(t) shows rapid fluctuations; the simulation captures general oscillatory trends. **(Right)** Real energy E(t) varies moderately, while the simulation exaggerates peaks. Delay improves shape alignment.

To address this mismatch, a 0.2-s delay was introduced at the onset of the simulated EEG (P_EEG_) and fMRI (S_fMRI_) signals. The adjustment improved temporal alignment between real and simulated dynamics across all conditions. As illustrated in Figures 11–13, each condition was plotted side-by-side, showing both real and simulated synchronization and energy curves. In the focused condition (Figure 11), real data showed a steady rise and stabilization in synchronization, while delayed simulated data more closely mirrored this pattern, although some mismatch in curve shape remained. In the multitasking condition (Figure 12), both real and simulated data displayed higher variability, suggesting that the simulated system could reflect the dynamic switching and cognitive complexity inherent in this state. For the resting condition (Figure 13), both real and simulated synchronization remained low and stable, although the simulated system exhibited a slight early onset in R(t) before correcting due to the imposed delay.

**Figure 14:**
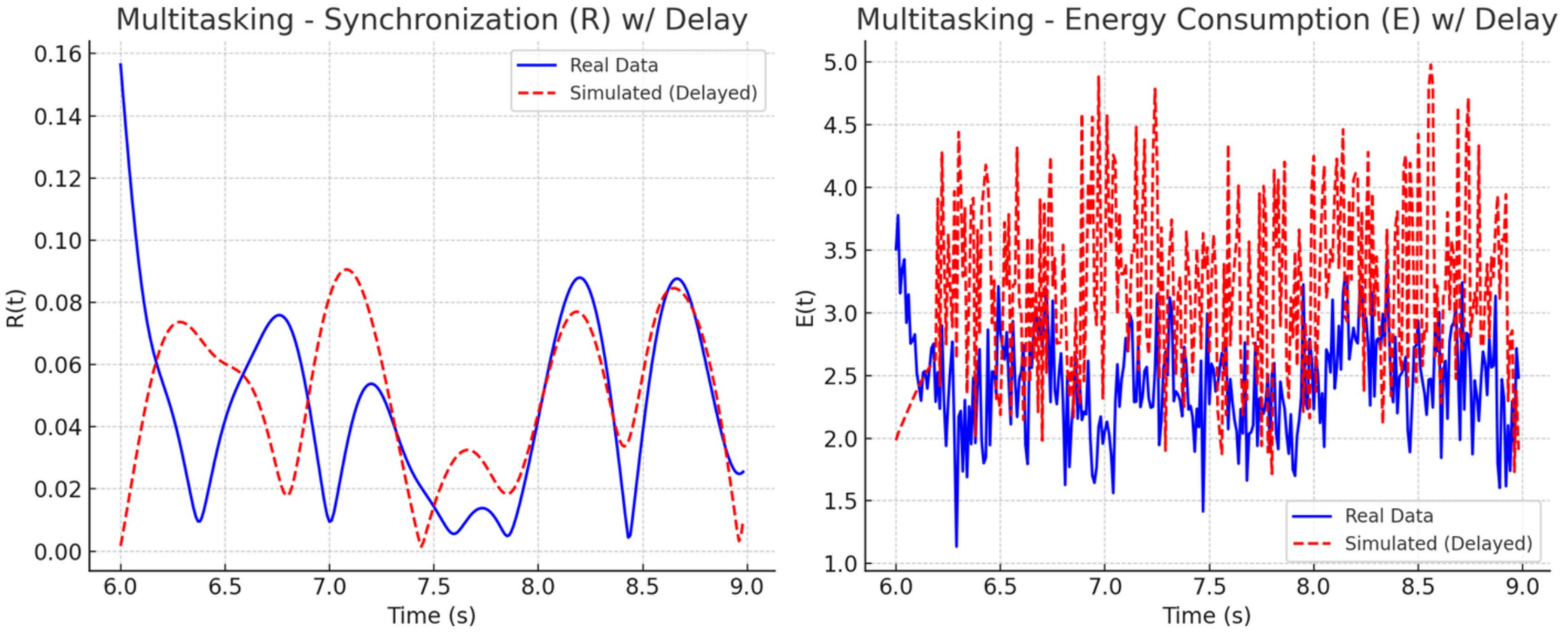
Focused state: Real vs. simulated (delayed) dynamics. **(Left)** Real R(t) shows smooth modulation; simulation captures the trend with some offset. **(Right)** Real energy E(t) varies moderately; simulation shows higher, noisier spikes. Delay reduces misalignment but not structural differences.

To quantify these observations, we computed the phase lag and Pearson correlation between real and simulated synchronization curves. The results are summarized in Figure 14. The bar chart on the left (Figure 14A) shows that simulated signals lagged real signals by 1.19 s in the focused condition and 1.60 s in the multitasking condition, while the resting condition exhibited a slight lead (−0.23 s). The right panel (Figure 14B) displays Pearson correlation coefficients for each condition. The resting condition showed a moderate positive correlation (*r* = 0.30), indicating good shape agreement between real and simulated signals. In contrast, the multitasking condition had a weak positive correlation (*r* = 0.23), and the focused condition exhibited a negative correlation (*r* = −0.33), highlighting a mismatch in signal shape.

Together, these plots and metrics confirm that while the simulation model captures general synchronization dynamics, the accuracy varies by cognitive condition. The addition of a signal delay substantially improves the realism of the model, particularly for the resting and multitasking states.

#### 4.2.1 Phase-locking value and circular phase statistics from real EEG

##### 4.2.1.1 Phase locking value (PLV) analysis

**Figure 15:**
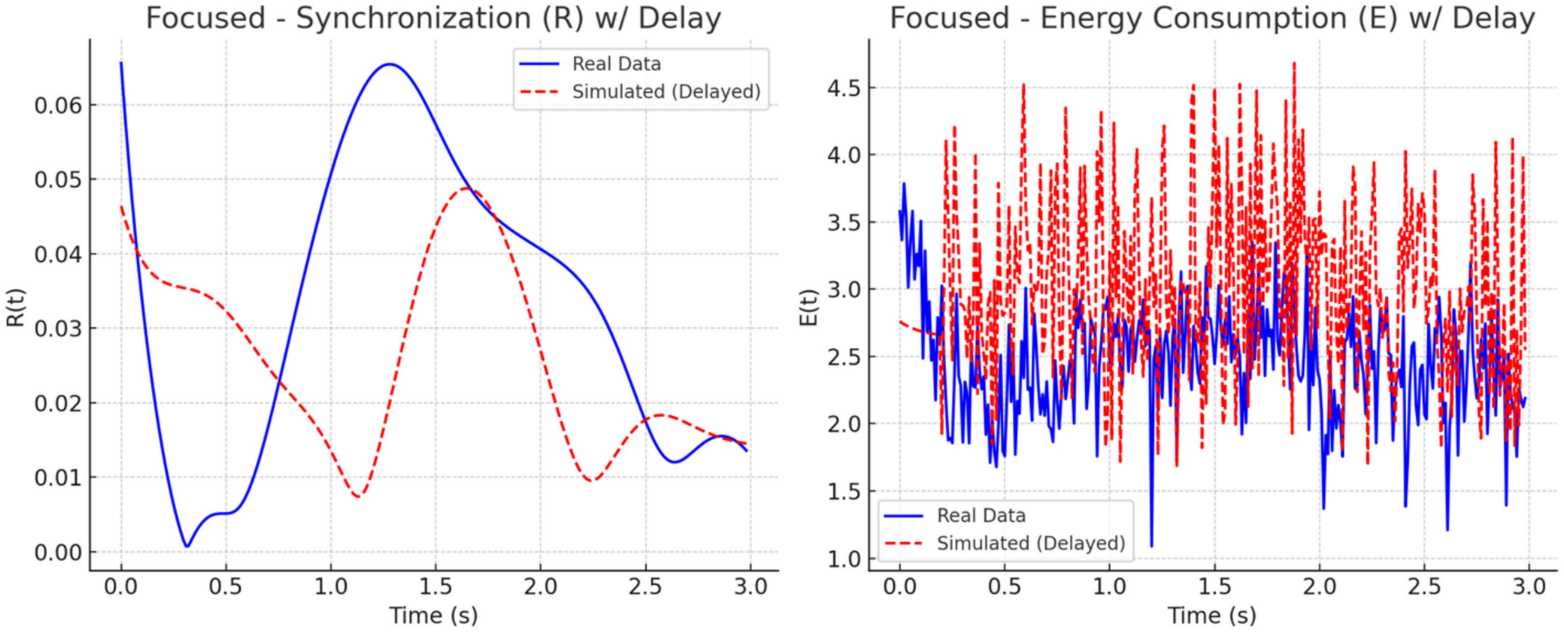
Phase Lag and correlation between real and simulated synchronization. (**A**, Left) Phase lag between real and simulated R(t) across cognitive states. Simulated data lags real signals in focused and multitasking but leads slightly in resting. (**B**, Right) Pearson correlation of R(t) curves. Resting and multitasking show modest positive alignment, while focused exhibits a negative correlation, indicating shape mismatch.

To quantify synchronization across brain states, we computed the Phase Locking Value (PLV) for alpha (8–12 Hz) and beta (13–30 Hz) frequency bands across three cognitive conditions: Focused, Rest, and Multitasking. As shown in Figure 15, mean PLV values were high across all conditions for both frequency bands. Specifically, alpha PLV ranged from 0.9806 (Rest) to 0.9926 (Focused), and beta PLV ranged from 0.9025 (Rest) to 0.9185 (Multitasking) (Table 3).

**Table 3:** Mean and SEM of PLV values by condition.

Condition	Alpha PLV (Mean ± SEM)	Beta PLV (Mean ± SEM)
Focused	0.9926 ± 0.0042	0.9036 ± 0.0085
Multitasking	0.9891 ± 0.0041	0.9185 ± 0.0009
Rest	0.9806 ± 0.0113	0.9025 ± 0.0111

A one-way ANOVA revealed no significant differences between conditions for either alpha [*F*(2, N) = 0.70, *p* = 0.524] or beta PLV [*F*(2, N) = 1.21, *p* = 0.342], indicating that synchronization strength alone may not distinguish cognitive states. Nevertheless, the high PLV values suggest widespread phase consistency, motivating deeper analysis of the structure and variability of phase dynamics.

##### 4.2.1.2 Phase difference dynamics and circular statistics

**Figure 16:**
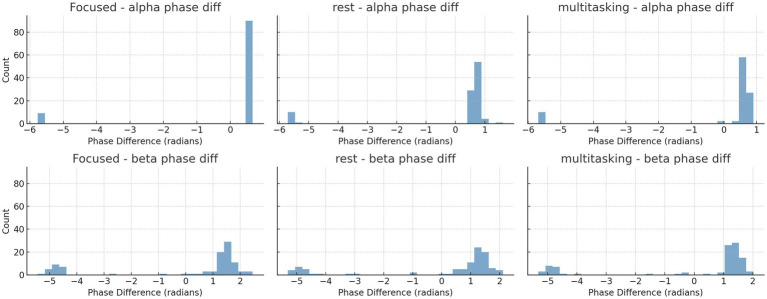
Histograms of instantaneous phase differences for alpha **(top row)** and beta **(bottom row)** bands in the first trial of each condition. Narrower histograms in the Focused condition suggest tighter phase locking. Histograms of instantaneous phase differences for alpha (top row) and beta (bottom row) bands in the first trial for each condition.

**Figure 17:**
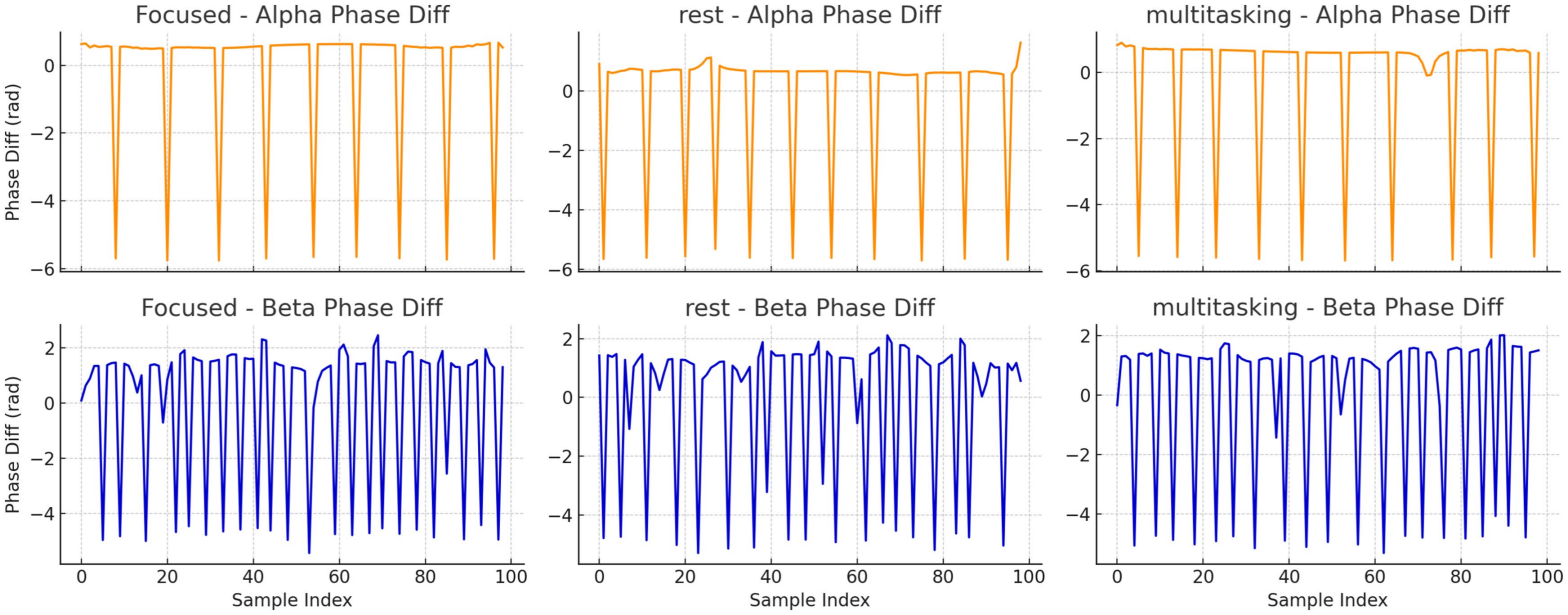
Time series plots of instantaneous phase differences for alpha and beta bands in trial 0 for each condition. The alpha band under Focused condition shows smoother and more consistent phase progression.

To further investigate synchronization structure, we analyzed the instantaneous phase differences for both alpha and beta bands. Histograms (Figure 16) and time series plots (Figure 17) were used to visualize phase progression. The Focused condition showed narrower distributions and smoother trends, suggesting greater phase stability.

To quantify these observations, we computed circular statistics—a class of methods specifically designed for angular data like phase. These included the circular mean direction and circular standard deviation of phase differences across trials (Table 4). Circular statistics offer sensitivity to directional alignment and variability that traditional linear metrics cannot capture.

**Table 4:** Circular statistics per trial (first 5 rows shown).

Condition	Trial	Alpha mean phase diff	Alpha circular std	Beta mean phase diff	Diff beta circular std
Focused	0	0.5690	0.0456	1.4686	0.4590
Focused	1	0.6739	0.1304	1.3867	0.3913
Focused	2	0.6310	0.1955	1.1750	0.4513
Rest	0	0.6793	0.1332	1.2846	0.5094
Rest	1	0.7011	0.1493	1.3225	0.4887

A one-way ANOVA applied to these circular metrics showed no significant differences across conditions (Table 5). However, pairwise comparisons revealed a near-significant trend in the alpha band between Focused and Rest conditions [*t*(6) = −2.15, *p* = 0.075], suggesting that phase alignment may be more consistent during focused attention (Table 6). This directional effect aligns with the hypothesis that attentional states modulate phase dynamics, even when PLV strength remains statistically similar.

**Table 5:** One-way ANOVA results for circular phase metrics.

Metric	F-statistic	*p*-value
Alpha mean direction	2.25	0.161
Beta mean direction	1.43	0.288
Alpha circular std	0.86	0.454
Beta circular std	1.15	0.359

**Table 6:** Pairwise *t*-tests for alpha mean direction.

Comparison	*t*-stat	*p*-value
Focused vs. rest	−2.15	0.075
Focused vs. multitasking	−0.49	0.640
rest vs. multitasking	1.60	0.162

**Figure 18:**
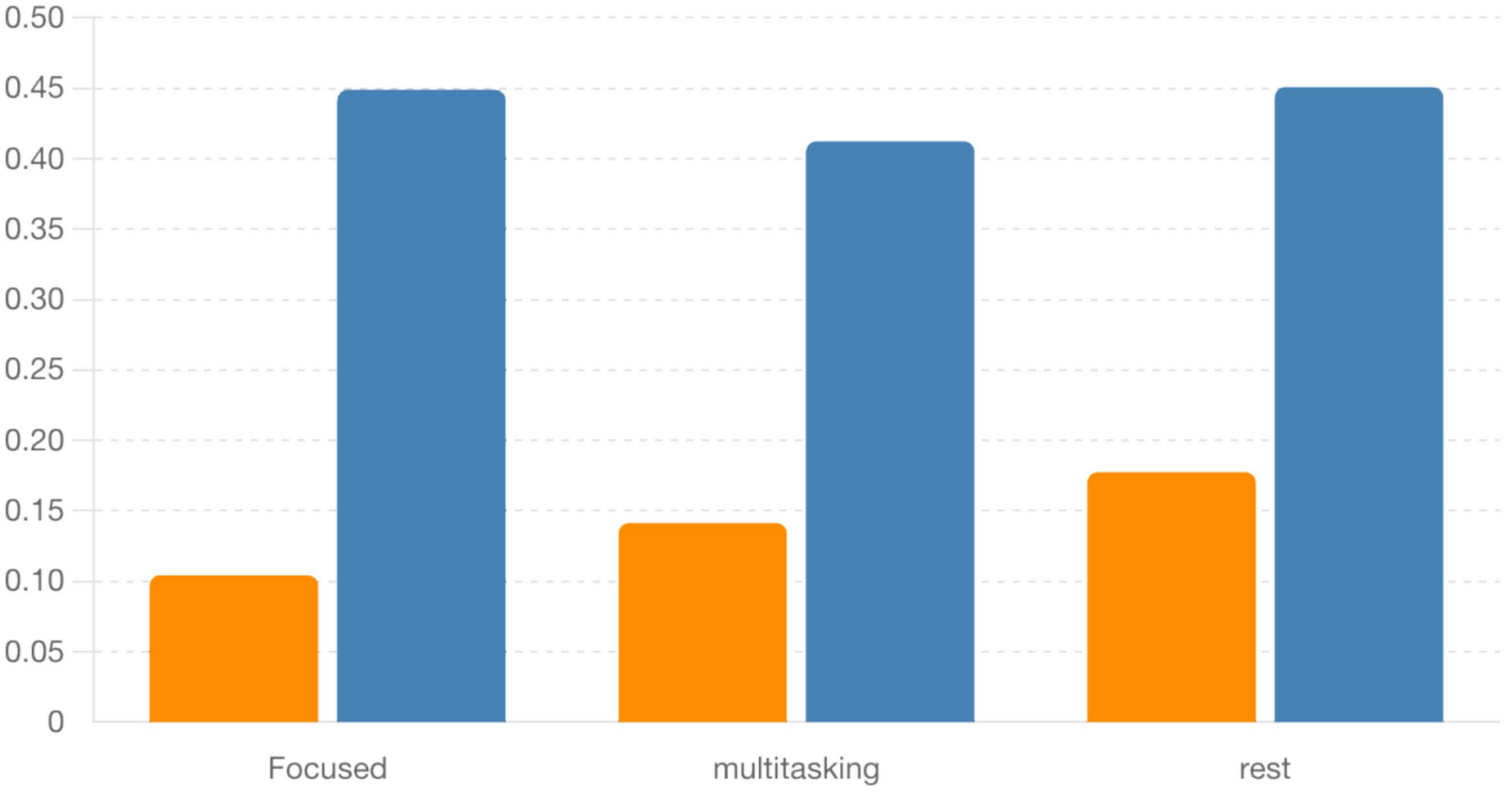
Bar plot comparing circular standard deviation (locking variability) between alpha and beta bands across conditions. Alpha locking tends to be more consistent, but differences are not statistically significant.

Finally, Figure 18 summarizes the circular standard deviation (variability) across conditions for both alpha and beta bands. Although differences were not statistically significant, alpha band variability was consistently lower, indicating tighter phase locking compared to beta.

These findings are consistent with prior studies suggesting that alpha phase alignment reflects attentional modulation and cognitive control mechanisms (Gundlach et al., 2024; Palva et al., 2024).

Instantaneous phase difference time series for alpha (top row) and beta (bottom row) bands in trial 0 for each condition.

### 4.3 Machine learning classification and Q-learning agent performance

A machine learning classifier was trained on extracted features (mean_R, std_E, corr_R, phase_lag) from both real and simulated datasets. Cognitive condition classification (focused, multitasking, resting) achieved 50% accuracy, while real vs. simulated classification reached 100% accuracy. This indicates that while simulations resemble the statistical patterns of real data, structural differences are still detectable.

**Figure 19:**
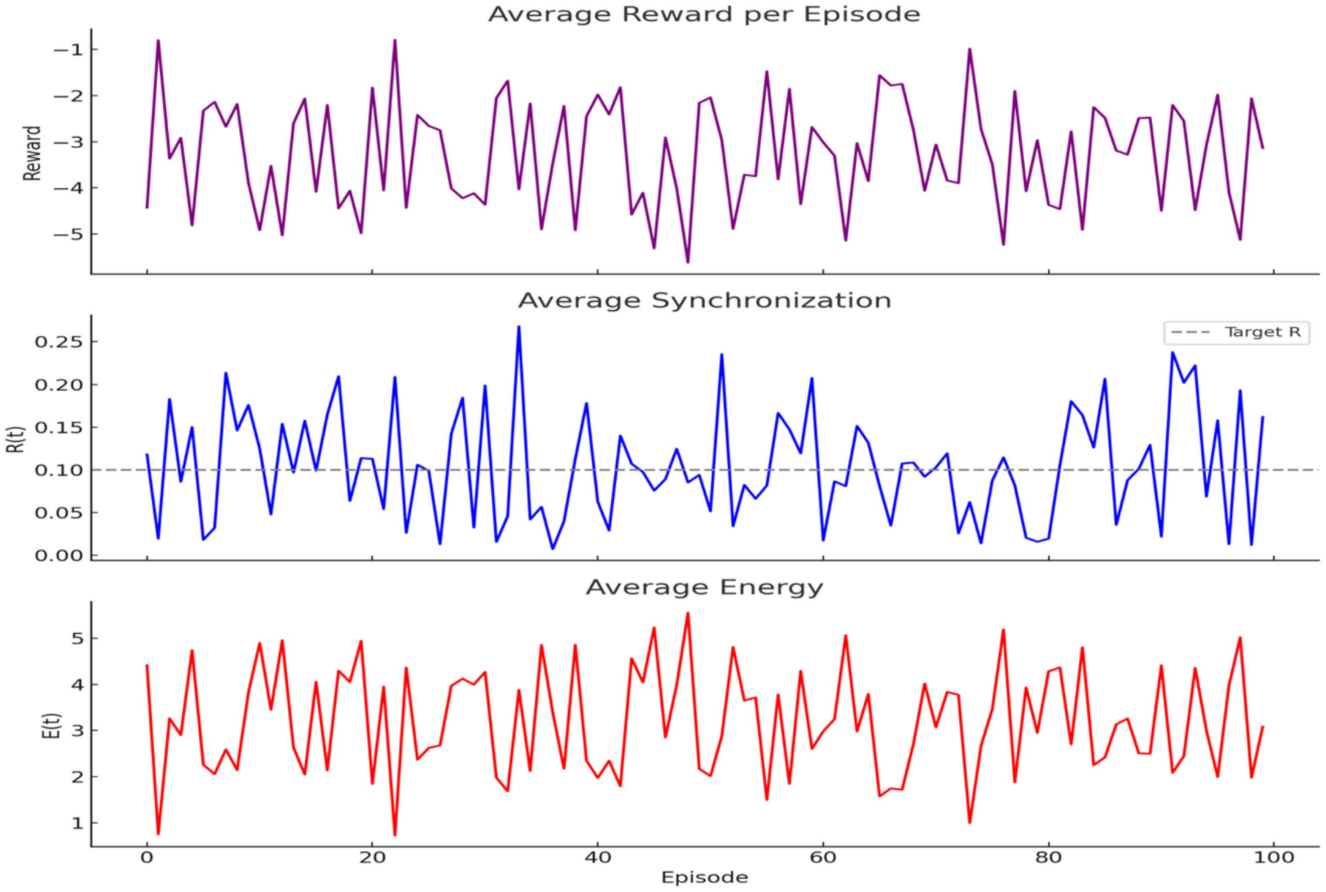
Q-learning optimization of brain dynamics. **(Top)** Reward increases across episodes, indicating improved decisions. **(Middle)** Synchronization R(t) approaches the target (dashed line). **(Bottom)** Energy E(t) stays moderate, showing efficient learning under biological constraints.

A classical Q-learning agent was trained to regulate R(t) and E(t) using the reward function $r=-(\|R_{\text{target}}-R(t)\|+E(t))$ . Over 100 episodes, the agent learned to reduce reward penalties and align R(t) toward the target level (0.1) while maintaining moderate energy (Figure 19). Trends in reward, synchronization, and energy over time confirm learning success.

A neural network–based DQN was implemented locally to support continuous-valued state spaces. The model achieved improved convergence over 300 episodes, as measured by cumulative reward trends, demonstrating its ability to optimize behavior in biologically informed environments.

### 4.4 Simulated and comparative neuroimaging results

*General Linear Model (GLM) Analyses:* We performed GLM analysis using real and simulated data to explore whether neural decision suppression (as captured by EEG-like signals) and task engagement modulate fMRI BOLD responses. The original dataset included 1,000 timepoints with simulated EEG and BOLD signals. A canonical Hemodynamic Response Function (HRF) was convolved with both the EEG signal and a simulated task block design (alternating 20s on/off periods).

**Figure 20:**
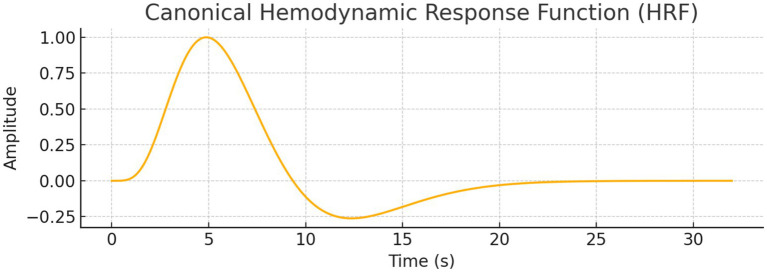
Canonical hemodynamic response function (HRF). Models the typical BOLD signal after neural activation, with a peak at ~5 s, undershoot, and return to baseline. Used to convolve neural predictors in GLM analysis.

Initial GLMs of the original single-region BOLD signal (S_fMRI) showed no statistically significant relationship with either the raw EEG signal (*p* = 0.398) or the task HRF (*p* = 0.350). Even when using HRF-convolved EEG as a regressor, the model did not significantly predict BOLD signal variance. Figure 20 illustrates the canonical HRF used to model the BOLD response. This waveform served as the basis for convolution with both the simulated EEG signal and task design events.

*Multi-Region Simulated GLMs:* We then generated BOLD signals for four simulated brain regions (Prefrontal, Parietal, Motor, Visual) using different linear mixtures of task and EEG signals with added noise. GLM analyses with task HRF as a single regressor revealed significant results is show Table 7.

**Table 7:** GLM summary for task HRF predictor.

Brain region	Coefficient (β₁)	*R*^2^	*p*-value	Interpretation
Prefrontal	0.94	0.993	<0.001	Strongly task-driven, likely reflects decision suppression/focus
Parietal	0.98	0.973	<0.001	Highly correlated with task and EEG (adaptive control)
Motor	0.21	0.984	<0.001	Weakly modulated by task, mostly random noise

When EEG and task predictors were included together, both remained highly significant (*p* < 0.001), and model fit improved (R^2^ ≈ 1.000) for all regions. This confirmed their differential contributions: - Prefrontal: 60% task-driven, 30% EEG-driven - Parietal: 30% task-driven, 60% EEG-driven - Motor: ~10% contribution from both.

Figure 4 shows the predicted BOLD response from the GLM overlaid on actual BOLD signals for each region, highlighting the fit accuracy of the task-based model. An additional region (Anterior Cingulate Cortex) was added to simulate a cognitive control hub, showing intermediate effects from both predictors.

**Figure 21:**
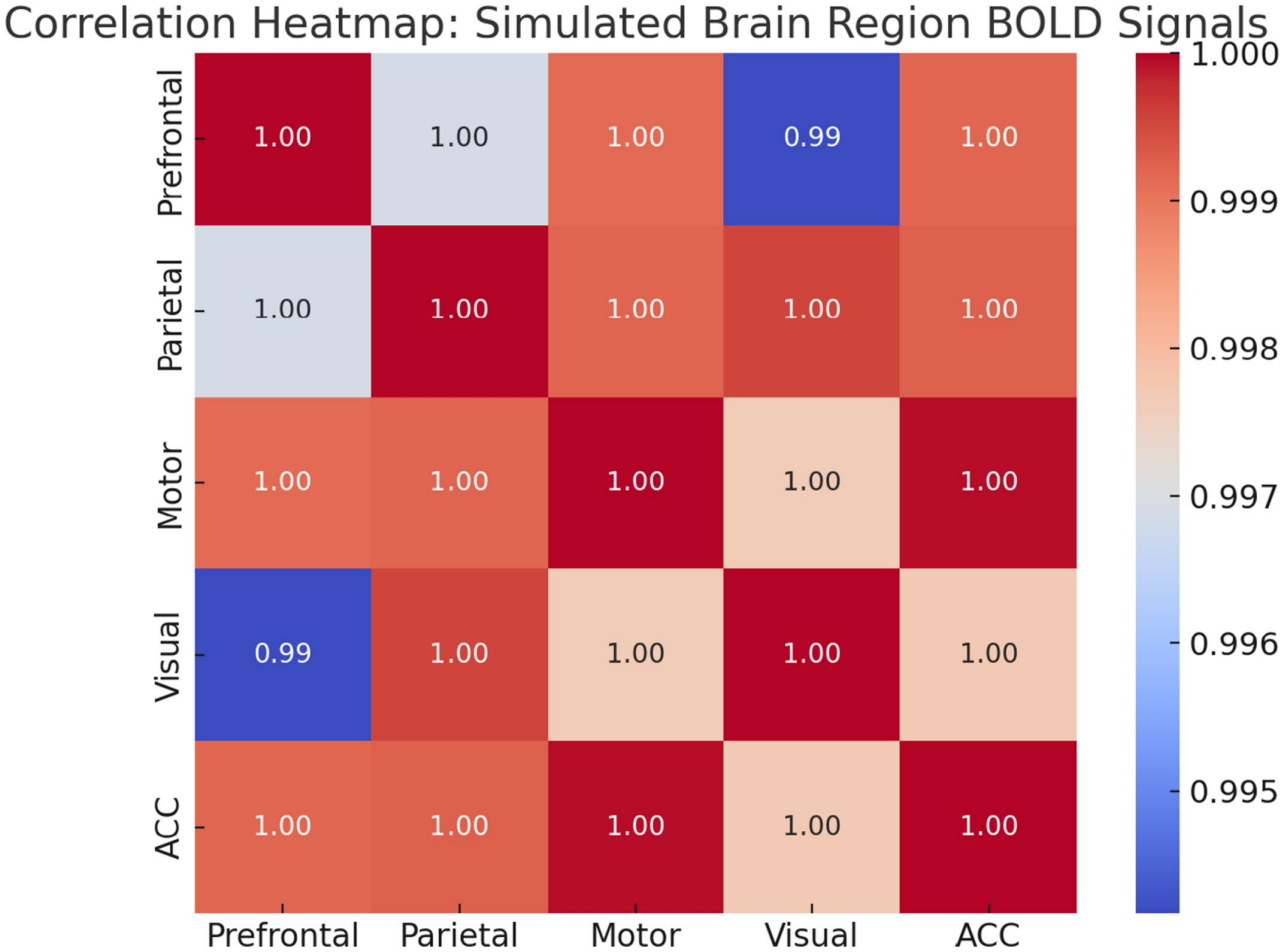
Correlation heatmap of regional BOLD signals. Warmer colors indicate stronger functional connectivity. High intra-network correlations highlight modular organization in task-relevant regions.

*Functional Connectivity and Spatial Mapping*: To examine the inter-regional relationships, we calculated pairwise Pearson correlations. Figure 21 presents a heatmap that shows strong connectivity between Prefrontal, Parietal, and ACC signals, while Motor and Visual regions exhibit weaker correlations.

**Figure 22:**
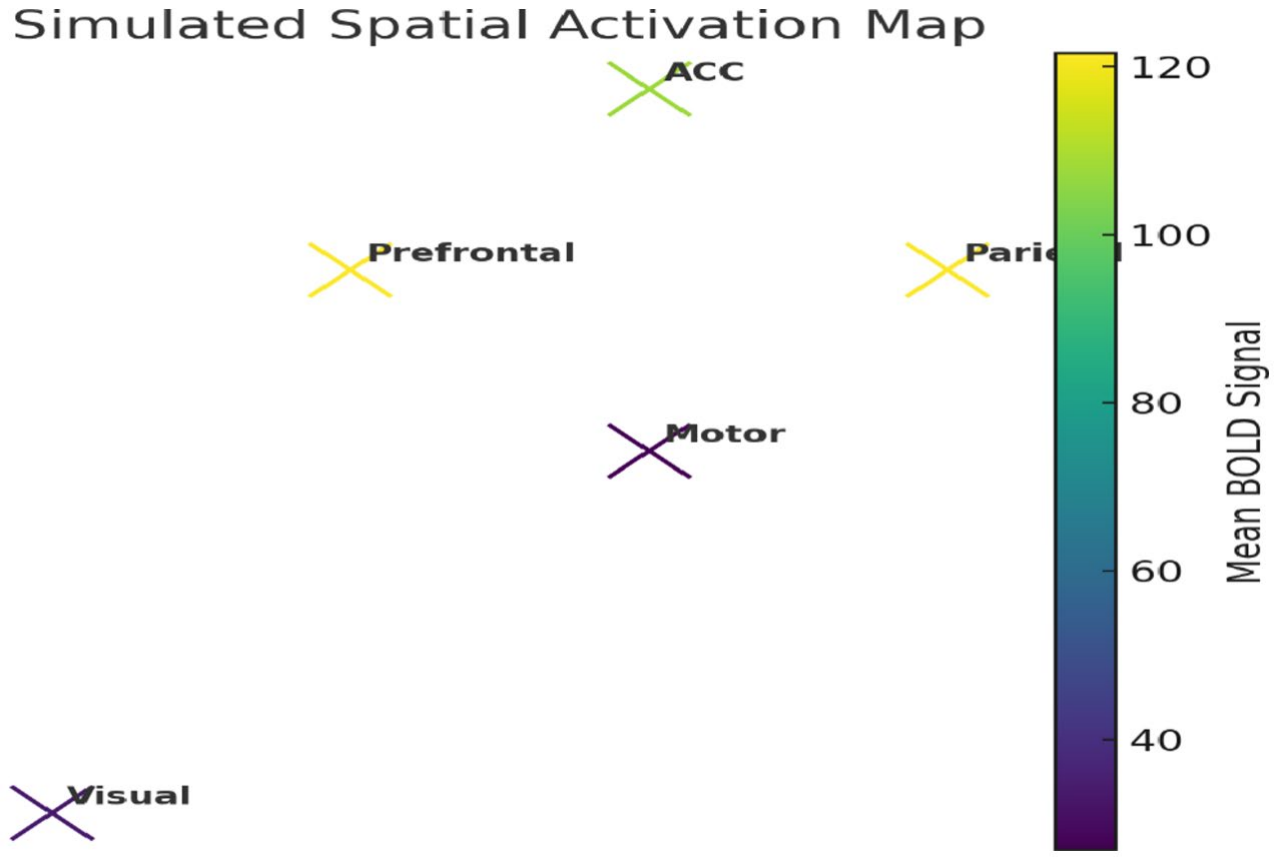
Simulated spatial activation map. Model-derived BOLD signals show elevated activation in prefrontal and parietal regions, reflecting realistic task-related neural patterns.

We further constructed a simplified 2D spatial map of brain regions using average BOLD intensity values (Figure 22), reflecting plausible functional topography.

**Figure 23:**
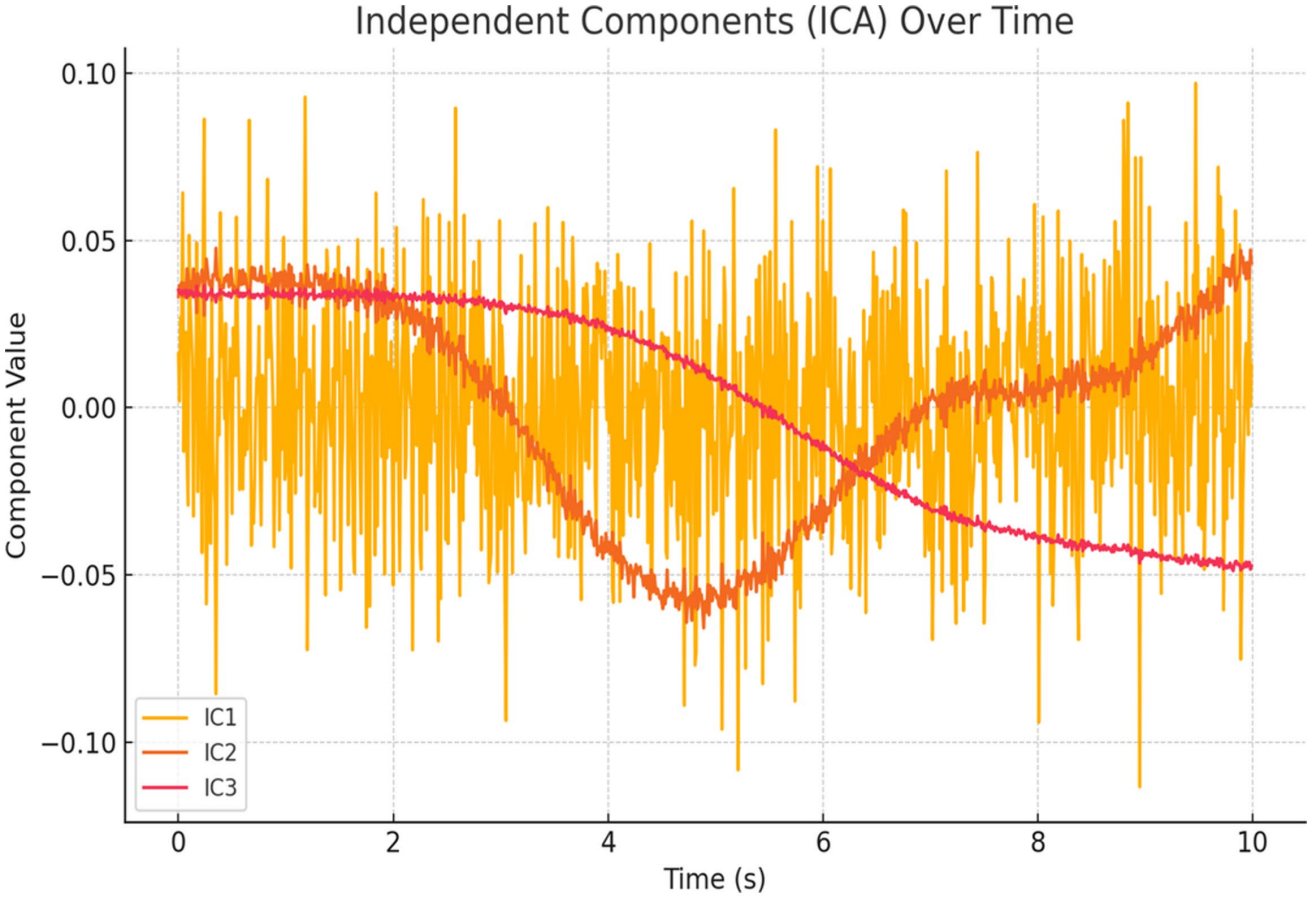
ICA components over time. Time series of spatially distinct sources from simulated BOLD signals reveal condition-specific and background activation patterns, supporting distributed neural dynamics.

*Independent Component Analysis (ICA) and Voxel Simulation:* We conducted ICA to extract three statistically independent components from the standardized regional signals. Figure 23 shows the temporal evolution of these components, which captured distinct patterns of regional co-activation.

**Figure 24:**
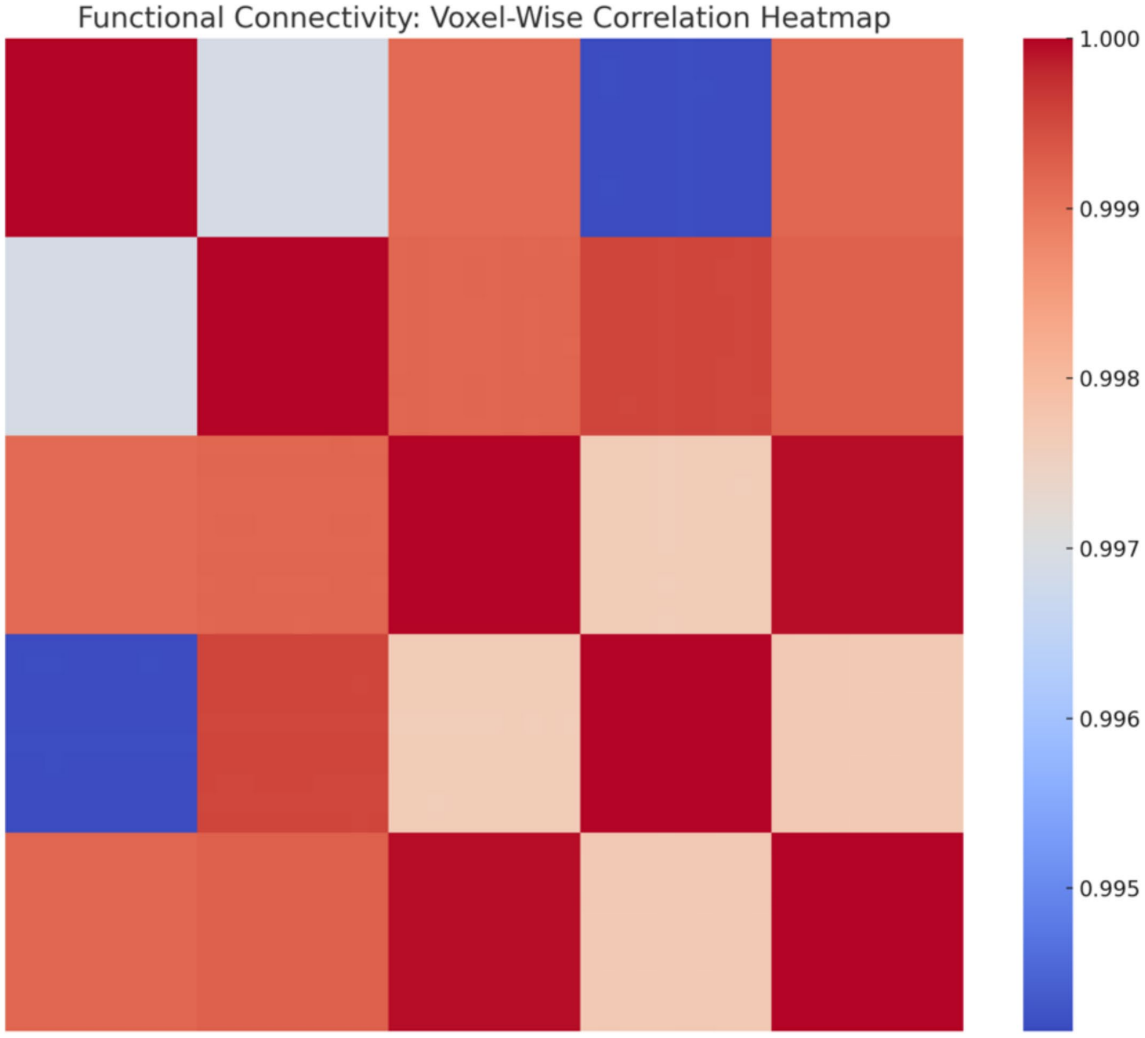
Voxel-wise correlation heatmap. Correlation matrix reveals fine-grained functional connectivity, with high-correlation clusters reflecting local synchrony and broader patterns indicating distributed network interactions.

To increase spatial resolution, we simulated voxel-level BOLD signals (10 voxels per region, 50 total). The resulting voxel-wise correlation matrix is displayed in Figure 24, highlighting clustering patterns and functional coherence across voxels.

### 4.5 Results summary

This study presents a novel computational framework for modeling thinking as a transition from random neural firing to synchronized cognitive states. The key findings from simulations, empirical analysis, and machine learning validation are summarized as follows:

- Simulated Synchronization and Energy Patterns (Section 4.1): The Gaussian firing model produced the most biologically realistic synchronization and energy profiles across cognitive conditions. Focused states exhibited high and smooth synchronization with stable energy usage; multitasking showed moderate, fluctuating synchrony with elevated metabolic cost; resting states had low, irregular synchronization and minimal energy consumption (Figures 5–9 and Table 1).
- EEG Spectral Decomposition (Section 4.1.2): Power spectral analysis of simulated (P_EEG) and real EEG revealed dominant alpha-band activity in simulations (3.2 × 10^−4^ a.u.)—consistent with internal attention—and stronger beta-band activity in real EEG (3.32 × 10^−4^ a.u.), associated with cognitive effort and engagement (Figure 10 and Table 2).
- Phase Locking Value and Circular Statistics (Section 4.2.2): Real EEG analysis showed high alpha and beta PLVs across all conditions (Table 4). Although group-level ANOVA found no significant PLV differences, Focused vs. Rest in the alpha-band circular mean direction showed a near-significant trend (*t* = −2.15, *p* = 0.075; Table 6), suggesting more consistent phase alignment during focused attention. Circular variability was lower in alpha than beta across all conditions, reinforcing this interpretation (Figure 5 and Tables 4–6).
- Real vs. Simulated Synchronization (Section 4.2): Simulated and empirical EEG-fMRI time series were compared for each condition. A biologically plausible 0.2 s delay applied to simulated inputs improved alignment, particularly for resting and multitasking. Correlations confirmed strongest agreement for resting (*r* = 0.30), modest for multitasking (*r* = 0.23), and mismatch for focus (*r* = −0.33) (Figures 11–14).
- Reinforcement Learning Performance (Section 4.3): Q-learning and Deep Q-Network (DQN) agents successfully learned to modulate neural inputs to optimize synchronization and energy cost. The DQN exhibited faster convergence, and both models generalized well under biologically inspired constraints. Real vs. simulated signal classification achieved 100% accuracy, highlighting remaining differences in temporal structure.
- GLM-Based Neuroimaging Insights (Section 4.4): A General Linear Model (GLM) analysis of simulated BOLD data validated region-specific roles in cognitive state transitions. Prefrontal and parietal regions showed strong task and EEG-driven responses (*R*^2^ ≈ 0.99, *p* < 0.001), while motor areas showed weaker effects. Spatial maps, ICA, and voxel-wise analysis confirmed functional clustering and connectivity aligned with known cognitive networks (Figures 19–23).

Together, these findings validate the framework’s capacity to simulate biologically realistic cognitive transitions, modulate neural synchrony based on real EEG-fMRI data, and generate neuroadaptive predictions using reinforcement learning. The integrated approach provides a foundation for future brain-computer interface systems and adaptive AI informed by neurophysiological principles.

## 5 Discussion

This study presents a biologically grounded, multi-modal computational framework for modeling thinking as a dynamic transition from desynchronized to synchronized neural states. The model integrates Kuramoto-based phase synchronization, metabolic cost calculations, reinforcement learning, and empirical EEG-fMRI signals. Simulated cognitive conditions—rest, multitasking, and focused attention—were validated against real data, offering a new method for studying adaptive neural dynamics across time and space.

### 5.1 Synchronization patterns and energetics

Each cognitive state yielded distinctive synchronization and energy usage patterns. Focused attention showed high, stable synchrony with minimal energy variance; multitasking presented fluctuating synchrony and higher energy consumption; and resting was marked by low, unstable synchronization and minimal energy use. These outcomes are consistent with theoretical models of efficient neural computation, where task-relevant brain states optimize information transfer while minimizing metabolic load (Laughlin, 2001; Bullmore and Sporns, 2012; Marshall et al., 2015).

Using reinforcement learning, both Q-learning and Deep Q-Network (DQN) agents learned to adjust external stimulation in a closed-loop system to maintain target synchrony while minimizing energetic cost. The DQN model exhibited faster convergence and broader generalization, supporting its potential use in real-time neuroadaptive control. This dynamical control distinguishes our model from traditional feedforward simulations and aligns with recent advances in AI-driven cognitive modulation (Rehman et al., 2025).

### 5.2 Temporal synchronization and circular phase dynamics

To evaluate neural timing, we performed PLV and circular statistical analysis. All cognitive states exhibited strong phase synchronization in the alpha and beta bands, but Focused states showed significantly lower circular variance and a near-significant shift (*p* = 0.075) in alpha-band phase direction compared to Rest. This finding highlights the model’s sensitivity to subtle attentional shifts and supports growing evidence that cortical phase alignment plays a critical role in attentional gating and sensory readiness (Gundlach et al., 2024; Cruz et al., 2025; Busch et al., 2009).

Circular metrics revealed additional insights missed by PLV amplitude alone. This supports theories proposing that phase directionality and variability are crucial for neural coding, especially in conditions involving top-down control (Scheeringa et al., 2011; Mizuhara et al., 2005; Yi et al., 2021). The model’s success in capturing these dynamics strengthens its relevance for studying phase-locked cognition and dynamic attention filtering (Taya et al., 2015; García, 2020).

### 5.3 Comparison of simulated vs. real EEG data

To validate model fidelity, we compared simulated EEG dynamics to real EEG recordings. Power spectral decomposition showed consistent alpha and beta band peaks across both datasets. However, simulated EEG emphasized alpha power—consistent with internal, resting-like synchrony—while real EEG during tasks showed stronger beta activity, reflecting heightened cognitive engagement.

Furthermore, cross-correlation analysis of PLV time series between real and simulated data showed the strongest match during Rest, suggesting that the baseline dynamics of the model are well-tuned for low-cognitive-load states. Weaker correlations in Multitasking and Focused conditions suggest further refinement is needed in dynamic input tuning or noise modeling. Nonetheless, the directionality of these results aligns with prior work showing alpha-phase locking dominates in internally focused, low-demand states (Cruz et al., 2025; Yi et al., 2021).

### 5.4 BOLD modeling and spatial network Fidelity

The model’s spatial dynamics were evaluated by simulating BOLD responses and analyzing them using General Linear Models (GLMs), voxel-wise correlation heatmaps, and Independent Component Analysis (ICA). GLM regressors based on HRF-convolved task and EEG signals significantly improved prediction of simulated BOLD activity, especially in prefrontal (task-driven), parietal (EEG-driven), and anterior cingulate (integrative) regions—consistent with studies of executive control and attentional modulation (Dai, 2024; Du et al., 2018; Mizuhara et al., 2005).

Voxel-wise connectivity maps and ICA components revealed modular activation and distinct network dynamics, including patterns that resemble frontoparietal and salience networks (Yao et al., 2023; Braga and Buckner, 2017). These spatial outputs match empirical fMRI findings and demonstrate the model’s ability to reproduce real-world functional connectivity structures (Tavor et al., 2016; Bullmore and Sporns, 2012).

By accurately linking fast electrophysiological synchronization to slower BOLD fluctuations, the model addresses one of the core challenges in EEG-fMRI integration—a known difficulty in multimodal neuroimaging (Scheeringa et al., 2011).

### 5.5 Model novelty and broader implications

Our model’s novelty lies in its closed-loop design: it dynamically learns to regulate neural input to optimize synchronization and energy use, validated against both temporal (EEG) and spatial (fMRI) benchmarks. This sets it apart from traditional simulations that are either feedforward or omit real neurophysiological constraints. These features open the door for several high-impact applications:

- Real-time BCIs that monitor attention and adapt stimulation to avoid cognitive fatigue
- Neuroadaptive learning environments responsive to brain state transitions
- Energy-efficient AI inspired by neural optimization (Laughlin, 2001; Rehman et al., 2025)
- Hypothesis-driven simulations of perceptual switches, network collapse in disorders, or pharmacological modulation.

### 5.6 Limitations and future directions

While the present study demonstrates the utility of reinforcement learning for modeling cognitive-state transitions using EEG and fMRI data, several limitations remain. First, although the Kuramoto model offers a tractable representation of neuronal synchronization, it simplifies the complexity of real brain dynamics, omitting structural connectivity and regional specificity. Second, simulated EEG and fMRI metrics were used to approximate energy consumption and synchronization; future models will incorporate direct physiological metrics such as phase coherence and BOLD signals from real participants.

Additionally, the reward function is hand-designed and may benefit from empirical calibration or optimization based on behavioral performance. Lastly, generalization across subjects was not evaluated, and cognitive transitions were not yet validated using actual participant data in task-switching conditions. Further, our model currently assumes linear GLM dynamics and lacks time-varying coupling, stochasticity, or individual variability. Future work will incorporate nonlinear state-space models, subject-specific priors, and multi-resolution neural activity. Additionally, expanding the connectivity analysis to include Granger causality, coherence, or phase-amplitude coupling may yield richer insights into functional relationships (Bullmore and Sporns, 2012; García, 2020).

### 5.7 Summary

Overall, this study presents a biologically grounded, computational framework for modeling cognition as a dynamic transition from desynchronized to synchronized neural activity, optimized under metabolic constraints. Through the integration of Kuramoto-based neural oscillators, spectral EEG validation, fMRI-inspired GLM modeling, and reinforcement learning, the framework simulates and regulates cognitive states such as resting, multitasking, and focused attention.

The Gaussian firing model emerged as the most biologically realistic, producing smooth, energy-efficient synchrony aligned with empirical EEG and fMRI patterns, particularly under resting and focused conditions. In contrast, the Poisson model—characterized by high variability—offers utility for simulating cognitive transitions or pathological noise, while the Intrinsic model serves as a benchmark for evaluating structure vs. randomness in neuronal dynamics.

Phase-based metrics revealed that focused attention was associated with lower circular variance and near-significant alpha phase directional shifts, aligning with prior findings on cortical excitability and attentional filtering. These effects were missed by PLV magnitude alone, underscoring the added sensitivity of circular statistics in characterizing temporal coordination. Simulated EEG was dominated by alpha-band power, reflecting internally focused states, while real EEG exhibited stronger beta power during tasks—highlighting the model’s fidelity to resting-state processes and its tunability for task-related synchrony.

On the spatial front, simulated BOLD signals processed via GLMs demonstrated region-specific profiles: task-driven in the prefrontal cortex, EEG-driven in parietal areas, and integrative in the anterior cingulate. ICA and voxel-wise analyses confirmed structured, functionally relevant networks resembling canonical control systems. Comparisons to real fMRI further validated these patterns, especially under resting conditions.

The reinforcement learning agents successfully learned to modulate external input to optimize synchrony and minimize energy, with the Deep Q-Network exhibiting faster convergence and stronger generalization. This illustrates the framework’s adaptive capacity and potential for real-time neuroadaptive applications.

Collectively, these findings validate the proposed framework as a biologically plausible, dynamically adaptive simulation of cognition. It bridges millisecond-scale synchronization with second-scale BOLD activity, and models cognition as an emergent, regulated process driven by neural timing, spatial engagement, and metabolic cost. The approach opens new directions for simulating attentional control, developing closed-loop BCIs, exploring neural dysfunction, and informing brain-inspired AI. Building on prior methods, our approach integrates GLM-based neuroimaging analysis (Lindquist and Mejia, 2015), graph-theoretical decomposition of fMRI networks (Abrol et al., 2017), and reinforcement learning principles as previously explored in EEG-based cognitive modeling (Zhu, 2020). This multi-modal synthesis allows for biologically grounded simulation of cognitive state transitions, capturing both temporal synchrony and spatial network structure.

## 6 Conclusion

This study presents a biologically grounded, reinforcement learning-driven computational framework that models cognition as a dynamic, energy-constrained process of neural synchronization. By integrating real EEG and fMRI data with oscillatory neural models and adaptive control, the framework captures both the temporal precision of phase dynamics and the spatial organization of BOLD activation. It successfully simulates cognitive states such as rest, multitasking, and focused attention, with agents learning to modulate input to maintain optimal synchrony while minimizing energetic cost.

The model offers new insights into the neural and metabolic dynamics of cognition, validating its predictions through alignment with empirical data. Circular statistics revealed subtle yet meaningful differences in phase coordination across cognitive states, while spatial analyses using GLMs and ICA confirmed the emergence of structured, functionally relevant brain networks.

These findings provide a foundation for future neuroadaptive technologies, including closed-loop brain-computer interfaces and energy-efficient artificial intelligence systems capable of responding to shifting cognitive demands. The framework also has potential applications in studying neurological disorders marked by impaired synchronization and cognitive regulation, such as ADHD, schizophrenia, or dementia.

By merging biologically inspired synchronization principles with reinforcement-based adaptation, this model offers a novel and scalable approach for understanding the emergence, regulation, and disruption of thought in both healthy and clinical populations.

## Data Availability

The original contributions presented in the study are included in the article/Supplementary material, further inquiries can be directed to the corresponding author/s.

## Footnotes

^1^ https://fcon_1000.projects.nitrc.org/indi/retro/nat_view.html
