# Supplementary material for: Modeling cognition through adaptive neural synchronization: a multimodal framework using EEG, fMRI, and reinforcement learning
Source: Front Comput Neurosci. 2025 Oct 16;19:1616472. doi: 10.3389/fncom.2025.1616472 (PMC12571814; doi:10.3389/fncom.2025.1616472)
Supplement: Supplementary file 1 [file Data_Sheet_1.pdf]

Appendix

Let's carefully show how to extract the phase dynamics from the coupled oscillator equations step by step. Starting from the complex amplitude equations and focusing on the phases $\phi_1(t)$ and $\phi_2(t)$, we aim to rewrite the equations for the phase difference $\phi = \phi_2 - \phi_1$.

Step 1: Start with the Amplitude Equations

From the coupled oscillator equations, the amplitude equations are:

$$2i\omega_1 \dot{A}_1 \approx \epsilon A_2 e^{i(\omega_2-\omega_1)}, \quad 2i\omega_2 \dot{A}_2 \approx \epsilon A_1 e^{i(\omega_1-\omega_2)} \tag{A.1}$$

Here, $A_1(t) = R_1 e^{i\phi_1(t)}$ and $A_2(t) = R_2 e^{i\phi_2(t)}$ are the complex amplitudes of the two oscillators, where:

- R_1 and R_2 are constant amplitudes (limit-cycle assumption),
- $\phi_1(t)$ and $\phi_2(t)$ are the time-dependent phases.

Step 2: Substitute A_1 and A_2

Substitute $A_1(t) = R_1 e^{i\phi_1(t)}$ and $A_2(t) = R_2 e^{i\phi_2(t)}$ into the equations.

For $\dot{A}_1$:

$$\dot{A}_1 = R_1 1 i\dot{\phi}_1 e^{i\phi_1}. \tag{A.2}$$

Substituting into the first equation:

$$2i\omega_1 R_1 i\dot{\dot{\phi}}_1 e^{i\phi_1} \approx \epsilon R_2 e^{i\phi_2} e^{i(\omega_2-\omega_1)t} - \epsilon R_1 e^{i\phi_1}. \tag{A.3}$$

For $\dot{A}_2$:

$$\dot{A}_2 = R_2 1 i\dot{\phi}_2 e^{i\phi_2}. \tag{A.4}$$

Substituting into the second equation:

$$2i\omega_2 R_2 i\dot{\dot{\phi}}_2 e^{i\phi_2} \approx \epsilon R_1 e^{i\phi_1} e^{i(\omega_1-\omega_2)t} - \epsilon R_2 e^{i\phi_2}. \tag{A.5}$$

Step 3: Simplify Using the Rotating Wave Approximation (RWA)

Assume the coupling is weak ($\epsilon \ll 1$) and that $\phi_1(t)$ and $\phi_2(t)$ vary slowly compared to the natural oscillations. Under this assumption, we neglect the fast oscillatory terms $e^{\pm i(\omega_1-\omega_2)t}$ in the equations.

1. For the first equation:

$$2\omega_1 R_1 \dot{\phi}_1 e^{i\phi_1} \approx \epsilon R_2 e^{i\phi_2} - \epsilon R_1 e^{i\phi_1} 1. \quad \text{(A.6)}$$

Divide through by $e^{i\phi_1}$ to isolate the terms involving ϕ_1:

$$2\omega_1 R_1 \dot{\phi}_1 \approx \epsilon R_2 e^{i(\phi_2-\phi_1)} - \epsilon R_1. \quad \text{(A.7)}$$

2. For the second equation:

$$2\omega_2 R_2 \dot{\phi}_2 e^{i\phi_2} \approx \epsilon R_1 e^{i\phi_1} - \epsilon R_2 e^{i\phi_2}. \quad \text{(A.8)}$$

Divide through by $e^{i\phi_2}$:

$$2\omega_2 R_2 \dot{\phi}_2 \approx \epsilon R_1 e^{i(\phi_1-\phi_2)} - \epsilon R_2. \quad \text{(A.9)}$$

Step 4: Extract Real and Imaginary Parts

Focus on the imaginary parts of the equations, as they govern the phase dynamics.

1. For the first oscillator:

Imaginary part:

$$2\omega_1 R_1 \dot{\phi}_1 \approx \epsilon R_2 sin\,(\phi_2 - \phi_1). \quad \text{(A10)}$$

Solve for $\dot{\phi}_1$:

$$\dot{\phi}_1 \approx \epsilon R_2 2\omega_1 R_1 sin\,(\phi_2 - \phi_1). \quad \text{(A.12)}$$

For the second oscillator:

Imaginary part:

$$2\omega_2 R_2 \dot{\phi}_2 \approx \epsilon R_2 sin\,(\phi_1 - \phi_2). \quad \text{(A.13)}$$

Solve for ϕ_2:

$$\dot{\phi}_2 \approx \epsilon R_1 \omega_2 R_2 sin\,(\phi_1 - \phi_2). \quad \text{(A.14)}$$

Step 5: Phase Difference Dynamics

Define the phase difference:

$$\phi = \phi_2 - \phi_1. \quad (A.15)$$

The time derivative is:

$$\dot{\phi} = \dot{\phi}_2 - \dot{\phi}_1. \quad (A.16)$$

Substitute ϕ˙1 and ϕ˙2 from above:

$$\dot{\phi} \approx \epsilon R_1 2\omega_2 R_2 si\,n(-\phi) - \epsilon R_2 2\omega_1 R_1 sin\,(\phi). \quad (A.17)$$

Using $sin\,(-\phi) = -sin\,(\phi)$:

$$\dot{\phi} \approx -(\epsilon R_1 2\omega_2 R_2 + \epsilon R_2 22\omega_1 R_1) sin\,(\phi). \quad (A.18)$$

For symmetric coupling ($R1 = R2$ and $\omega 1 = \omega 2$), this simplifies to:

$$\dot{\phi} = \Delta\omega - \epsilon sin\,(\phi), \quad (A.19)$$

where

$\Delta\omega = \omega_2 - \omega_1$ is the natural frequency detuning. (A.20)

Conclusion

The resulting equation for the phase difference ϕ is:

$$\frac{d\phi}{dt} = \Delta\omega - \epsilon sin\,(\phi). \quad (A.21)$$

This governs the synchronization behavior of the two coupled oscillators. If $|\ \Delta\omega\ |\leq \epsilon|$, the oscillators synchronize with a stable phase difference; otherwise, they remain desynchronized.
